# Microbiota metabolite butyrate constrains neutrophil functions and ameliorates mucosal inflammation in inflammatory bowel disease

**DOI:** 10.1080/19490976.2021.1968257

**Published:** 2021-09-08

**Authors:** Gengfeng Li, Jian Lin, Cui Zhang, Han Gao, Huiying Lu, Xiang Gao, Ruixin Zhu, Zhitao Li, Mingsong Li, Zhanju Liu

**Affiliations:** a Center for IBD Research, Department of Gastroenterology, Shanghai Tenth People’s Hospital, Tongji University School of Medicine, Shanghai, China; b Department of Bioinformatics, School of Life Sciences and Technology, Tongji University, Shanghai, China; c Division of Immunology, School of Basic Medical Sciences, Henan University of Science and Technology, Luoyang, China; d Department of Gastroenterology, Third Affiliated Hospital of Guangzhou Medical University, Guangzhou, China

**Keywords:** Inflammatory bowel disease, butyrate, neutrophils, neutrophil extracellular traps, inflammatory mediators

## Abstract

Host-microbial cross-talk plays a crucial role in maintenance of gut homeostasis. However, how microbiota-derived metabolites, e.g., butyrate, regulate functions of neutrophils in the pathogenesis of inflammatory bowel disease (IBD) remains elusive. We sought to investigate the effects of butyrate on IBD neutrophils and elucidate the therapeutic potential in regulating mucosal inflammation. Peripheral neutrophils were isolated from IBD patients and healthy donors, and profiles of proinflammatory cytokines and chemokines were determined by qRT-PCR and ELISA, respectively. The migration and release of neutrophil extracellular traps (NETs) were studied by a Transwell model and immunofluorescence, respectively. The *in vivo* role of butyrate in regulating IBD neutrophils was evaluated in a DSS-induced colitis model in mice. We found that butyrate significantly inhibited IBD neutrophils to produce proinflammatory cytokines, chemokines, and calprotectins. Blockade of GPCR signaling with pertussis toxin (PTX) did not interfere the effects whereas pan-histone deacetylase (HDAC) inhibitor, trichostatin A (TSA) effectively mimicked the role of butyrate. Furthermore, *in vitro* studies confirmed that butyrate suppressed neutrophil migration and formation of NETs from both CD and UC patients. RNA sequencing analysis revealed that the immunomodulatory effects of butyrate on IBD neutrophils were involved in leukocyte activation, regulation of innate immune response and response to oxidative stress. Consistently, oral administration of butyrate markedly ameliorated mucosal inflammation in DSS-induced murine colitis through inhibition of neutrophil-associated immune responses such as proinflammatory mediators and NET formation. Our data thus reveal that butyrate constrains neutrophil functions and may serve as a novel therapeutic potential in the treatment of IBD.

## Introduction

Inflammatory bowel diseases (IBD), composed of Crohn’s disease (CD) and ulcerative colitis (UC), are chronic relapsing inflammatory diseases in the gastrointestinal tract characterized by abdominal pain, diarrhea, and body weight loss.^1^ Although the precise etiologies and pathology are still unclear, it is generally considered that IBD is caused by a confluence of genetic and environmental factors that influence gut mucosal homeostasis to trigger dysregulated immune response to resident microbiota. In the past decades, it has witnessed the great advances in the basic and clinical studies on the pathogenesis of IBD. However, due to its intricate pathogenesis and huge interindividual heterogeneity, IBD still poses a great challenge to modern management and therapy. Thus, to clarify the immunopathogenesis of IBD clearly and discover novel therapeutics would provide support for precision medicine and individualized treatments in the future.

A delicate and ingenious balance between host and gut microbiota contributes to intestinal homeostasis. Accumulating lines of evidence have proven that gut microbiota-derived antigen-induced aberrant immune responses are initial events of chronic relapsing intestinal inflammation. Nevertheless, metabolites from resident beneficial bacteria eventually influence intestinal immune maturation and homeostasis. Alterations in the compositions of microbiota and its metabolite profiles have been demonstrated to be associated with the development of IBD.^2^ Multitudes of studies have elucidated that gut microbiota-derived metabolites participate in the maintenance of intestinal homeostasis as well as in the pathogenesis of IBD.^3^ Short chain fatty acids (SCFAs), with fewer than 6 carbon atoms, are by-products from gut microbiota fermentation of indigestible dietary fiber. Multiple lines of evidence have proved that SCFAs exert anti-inflammatory role and promote the integrity of epithelial barrier function.^4,5^ The principal SCFAs, including acetate, propionate and butyrate, constitute more than 95% of the total content of SCFA in the feces. Previous studies have indicated that there are two major mechanisms through which SCFAs take effects, including activation of GPCRs and inhibition of HDAC. The short chain fatty acid butyrate is found to provide energy resources for colonocytes and, meanwhile, upregulate Treg pool in the colonic lamina propria.^6,7^ SCFAs, especially butyrate, also promote Th1 cell production of IL-10 so as to maintain intestinal homeostasis.^8^ Furthermore, macrophages differentiated in the presence of butyrate also show enhanced antimicrobial functions and these effects are confirmed to be mediated by HDAC3 inhibition.^9^ Importantly, recent study has also demonstrated that butyrate promotes IL-22 production in CD4^+^ T cells and innate lymphoid cells (ILCs), leading to the protection of intestine from inflammatory damage.^10^

Numerous studies over the years have linked intestinal mucosal CD4^+^ T cells and the released pro-inflammatory cytokines to the pathogenesis of IBD.^11^ Increasing studies have focused more attentions on mucosal innate immune responses, such as epithelial barrier function, autophagy, and innate immune recognition. Innate immune cells including neutrophils, monocytes, and macrophages together constitute the innate barrier of phagocytes.^12^ Nowadays, more and more evidences have shown that neutrophils are also implicated in the development of chronic inflammatory diseases such as IBD. Neutrophils, as first responder of immune cells, are recruited into the inflammatory intestinal mucosa and eradicate invaded microbes through the phagocytosis, degranulation, and release of neutrophil extracellular traps (NETs).^13^

Previous studies have demonstrated that dysfunctions of neutrophils are associated with intestinal inflammation. Congenital disorders with single-gene defects such as glycogen storage disease type 1 and chronic granulomatous disease display abnormalities in neutrophil functions and cause IBD-like intestinal inflammation. Neutrophils from patients with CD display defective superoxide generation and phagocytosis,^14^ and we also demonstrated that CD177^+^ neutrophils, as one subset of functionally activated neutrophils in IBD patients, produce IL-22 to maintain epithelial integrity.^15^ Consistently, mice depleted of neutrophils show an exacerbated intestinal inflammation upon TNBS insult.^16^ Conversely, excessive recruitment and accumulation concomitant with delayed resolution of neutrophils have been accused to lead to persistent inflammatory activation and mucosal injury.^17,18^ Collectively, these studies point out that alterations of neutrophil functions play an important role in the pathogenesis of IBD, suggesting a promise of potentially modulating neutrophils in the management of disease. Although the underlying mechanisms whereby SCFAs modulate immune responses in different immune cells have been unmasked, how butyrate regulates functions of neutrophils in IBD patients is still poorly understood.

In the current study, we investigated potential roles of butyrate in modulating immune responses of neutrophils from IBD patients and explored the effects of butyrate on the induction of acute DSS colitis in mice. We found that butyrate significantly inhibited LPS-induced neutrophil production of proinflammatory cytokines, chemokines and calprotectin from IBD patients, and downregulated the release of MPO, ROS, and formation of NETs. Furthermore, intestinal mucosal inflammation was markedly relieved in DSS-induced colitic mice after feeding with butyrate, as characterized by decreased neutrophil-associated inflammatory mediators and NETs in intestinal mucosa. Therefore, our results demonstrate that butyrate effectively modulates functions of neutrophils and limits the proinflammatory potentials, thus ameliorating mucosal inflammation in IBD. These results provide a novel therapeutic potential of butyrate in treatment of IBD.

## Materials and methods

### Subjects and samples collection

All IBD patients or healthy volunteers from whom clinical samples were collected were recruited from Department of Gastroenterology, the Shanghai Tenth’s People’s Hospital of Tongji University (Shanghai, China) from November 2018 to May 2021. EDTA-anticoagulated blood samples (10–15 mL) were obtained from patients with active CD (A-CD, n = 37), patients with active UC (A-UC, n = 43), and healthy volunteers (HC, n = 32) after overnight fasting. The diagnosis of CD or UC was based on a combination of clinical presentation, endoscopy and radiological examination, and histological findings after exclusion of other diseases. Disease severity was evaluated according to CD activity index (CDAI) and Mayo scores for CD and UC, respectively. The demographics and disease characteristics of the patients in this study are summarized in Supplementary Table S1.

This current work was approved by the Institutional Review Board for Clinical Research of the Shanghai Tenth People’s Hospital of Tongji University. Written informed consent was obtained from all subjects before the study.

### Mice

Male C57BL/6J mice were purchased from Shanghai SLAC Laboratory Animal Co., Ltd (Shanghai, China), and bred and reared under specific pathogen-free facilities at the Experimental Animal Center of Tongji University School of Medicine (Shanghai, China). All mice could freely access to filtered air, sterile water, and autoclaved food in individually ventilated cages with a 12-h light-dark cycle. Mice used in this study were at 8 to 10 weeks of age and 20 to 25 g of body weight. All mouse experiments and procedures in this study were approved by the Institutional Animal Care and Use Committee of Tongji University.

### Reagents and antibodies

TRIzol reagents (Cat: 15596026), fluorochrome-conjugated anti-mouse CD11b (Cat: 17–0112-81) and live/dead cell viability assay kits (Cat: L34964 and L10119) were purchased from Invitrogen (San Diego, CA, USA). Fluorochrome-conjugated anti-mouse Ly6G (Cat: 560601) was purchased from BD Biosciences (San Diego, CA, USA). Sodium butyrate (C4; Cat: 303410), pertussis toxin (PTX; Cat: 516562), lipopolysaccharide (LPS; Cat: L4516), phorbol 12-myristate 13-acetate (PMA; Cat: P8139) were purchased from Sigma-Aldrich (St. Louis, MO, USA). Trichostatin A (TSA; Cat: S1045) was purchased from Selleck (Houston, TX, USA). Recombinant Human IL-8 protein (Cat: 208-IL-010/CF) was purchased from R&D systems (Minneapolis, MN, USA). DSS (36–50 kDa, Cat: 9011–18-1) was purchased from MP Biomedicals (Santa Ana, CA, USA). The following ELISA assay kits for human IL-6 (Cat: 430504), TNF-α (Cat: 430204), IFN-γ (Cat: 430101), IL-8 (Cat: 431504), S100A8/9 (Cat: 439707), lipocalin-2 (LCN2, Cat: 443407), and proinflammatory chemokine panel (CCL3, CCL4, CXCL1, Cat: 740987) were purchased from BioLegend (San Diego, CA, USA). Western blot antibodies against citrullinated histone H3 (Cat: ab5103) and β-actin were purchased from Abcam (Cambridge, UK) and Santa Cruz Biotechnology (Dallas, TX, USA), respectively. Secondary antibodies against rabbit (Cat: 7074) and mouse (Cat: 7076) antibodies were purchased from Cell Signaling Technology (Danvers, MA, USA).

### Preparation of human peripheral neutrophils

Neutrophils were collected from peripheral anticoagulated blood samples after density gradient centrifugation. Briefly, peripheral blood samples were firstly diluted with equivoluminal phosphate-buffered solution and then carefully layered on Ficoll-Paque^TM^ PLUS (GE Healthcare; Uppsala, Sweden) followed with gradient centrifugation at 2000 rpm for 30 min at 20°C. The bottom layer was collected to obtain neutrophils after hypotonic lysis of red blood cells with Lysing Buffer (BD Biosciences; San Diego, CA, USA) according to manufacturer’s instructions. Finally, neutrophils were resuspended in RPMI-1640 medium (Gibco, Life Technologies; Grand Island, NY, USA) supplemented with 100 U/mL penicillin, 100 µg/mL streptomycin, and 10% FBS for next use.

Neutrophil viability and morphology were determined by trypan blue exclusion test in a Neubauer counting chamber and observed under optical microscopy. Cell purity (≥95%) was confirmed by flow cytometric analysis before any of the assays was performed (Supplementary Figure S1).

### Assessment of neutrophil survival and apoptosis

Neutrophil survival was evaluated by using LIVE/DEAD™ Fixable Near-IR Dead Cell Stain Kit (Cat: L10119, Invitrogen) according to the manufacturer’s instructions. Neutrophils were isolated from healthy controls (n = 3) and treated with different concentrations of butyrate at several time points. Neutrophils were then collected and stained with live/dead dye and fluorochrome-conjugated anti-human antibodies against CD66b. Neutrophil apoptosis was evaluated by using an Annexin V-fluorescein isothiocyanate (FITC) Apoptosis Detection Kit (ebioscience; San Diego, CA, USA) according to the manufacturer’s instructions. Briefly, neutrophils were isolated from healthy controls (n = 3) and treated with TNF-α (20 ng/mL) in the presence or absence of indicated concentrations of butyrate for 3 h. Subsequently, cells were collected and stained with FITC-conjugated Annexin V and PI for 15 min at room temperature. Flow cytometric analysis was performed on BD FACS Canto II (BD Biosciences; San Diego, CA, USA) and analyzed by FlowJo VX software (Tree Star, Inc.; Ashland, OR, USA). The concentrations of butyrate used across the study have no impact on neutrophil survival or apoptosis (Supplementary Figures S2,3).

### Quantitative real-time polymerase chain reaction (qRT-PCR)

Total RNA of neutrophils was extracted with TRIzol reagent and the concentration and purity were also detected to assure the quality. RNA was then used as template for reverse transcription into complementary DNA (cDNA), which was performed by using a 5× All-in-one RT MasterMix Kit (Applied Biological Materials; Richmond, BC, Canada) based on the manufacturer’s instructions. RT-PCR conditions we used were as follows: 25°C for 10 min, 42°C for 15 min, and 85°C for 5 min, and qRT-PCR was performed in the 7900HT Fast Real-Time PCR system (Applied Biosystems; Carlsbad, CA, USA) using a TB Green Premix Ex Taq PCR Kit (TaKaRa; Dalian, China). The cycling conditions for qRT-PCR reactions were as follows: 30 s at 95°C, followed by 40 cycles of 5 s at 95°C and 30 s at 60°C. The relative expression levels of target gene were normalized to housekeeping gene, GAPDH, and calculated by the 2^–ΔΔCT^ algorithm. All primers utilized in this study were synthesized in Sango Biotech (Shanghai, China) and primer sequences are detailed in Supplementary Table S2.

### Enzyme-linked immunosorbent assay (ELISA)

The levels of proinflammatory mediators in the culture supernatants of neutrophils were measured by ELISA kits. Briefly, capture antibodies were added and incubated in 96-well plate overnight at 4°C. On next day, assay diluent was used to block nonspecific binding and reduce background. After incubation with standards or sample proteins at room temperature for 2 h with gently shaking, the plates were incubated with detection antibody for 1 h and avidin-HRP for 30 min in succession. Finally, TMB substrate and stop solution were added to develop the color. The absorbance was read at 450 nm with a microplate spectrophotometer (BioTek; Winuschi, VT, USA).

### Neutrophil transmigration assay

Assessment of neutrophil transmigration was conducted by using Hanging cell culture inserts (3 μm) (Millicell; Billerica, MA, USA). Briefly, neutrophils (5 × 10^5^/well) stimulated with or without butyrate (0.5 mM) were seeded onto the inside of the insert above the transmigration membrane and tissue culture media dissolved with IL-8 (20 ng/mL) were added to basolateral side of each well. After incubation for 2 h, transmigrated cells on the membrane were fixed in 4% paraformaldehyde (PFA) and then stained with 0.1% crystal violet staining solution (Sangon Biotech; Shanghai, China). Finally, the images were observed by optical microscopy.

### Detection of neutrophil production of reactive oxygen species (ROS)

Effects of butyrate on neutrophil production of ROS were evaluated as follows. Isolated neutrophils were firstly incubated with butyrate (0.5 mM) as indicated for 3 h and then stimulated with PMA (100 ng/mL) for 30 min. The levels of ROS in the culture supernatant were measured by using Amplex Red Hydrogen Peroxide Assay Kit (Invitrogen) according to the manufacturer’s instructions. The OD value was determined by microplate spectrophotometer (BioTek; Winuschi, VT, USA).

### Induction of NETs

Round coverslips were coated with poly-L-lysine (0.5 mg/mL) in advance for adherence of neutrophils. The experiments were carried out using a 24-well sterile culture plate with poly-L-lysine-coated coverslips. Neutrophils were preincubated with or without butyrate (10 mM) for 30 min and then stimulated with PMA (100 ng/mL) for 3 h to induce NETs. Finally, adherent neutrophils were fixed in 4% PFA and incubated with Hoechst 33342 (1:1000) to stain the decondensed chromatin network.^15^ Photographs of all specimens were observed under fluorescence microscopy (AF6000, Leica; Wetzlar, Germany).

### Establishment of acute DSS-induced colitis in mice

Acute DSS-induced colitis was established in mice as described previously.^15^ Briefly, C57BL/6 mice were administered with 2.0% DSS in drinking water for 7 days followed by 3 days of normal drinking water. Mice were also fed butyrate (200 mM) in drinking water during the entire course. Mice were monitored daily for diarrhea, weight change and rectal bleeding. All mice were sacrificed on day 10, and the colon tissues were removed and processed to quantify the numbers of neutrophils infiltrated in colons. The distal portions of colon were obtained for HE and immunohistochemical staining, and RNA and protein extraction.

### Immunofluorescence staining of NETs in colon tissues

The distal colon tissues of DSS-induced colitic mice were collected, fixed with 4% paraformaldehyde and embedded in a paraffin block followed by cutting into 5 μm slices. These sections were then transferred onto glass slides for next use. After deparaffinization and rehydration, these slides were treated with 0.3% Triton X-100 for 10 min at room temperature and blocked with 10% normal donkey serum for 1 h. Subsequently, immunostaining was performed with rabbit anti-mouse Citrullinated H3 (CitH3; ab5103, Abcam) and rat anti-mouse Ly6G (ab25377, Abcam) overnight at 4°C. After incubated with secondary antibodies coupled with Alexa Fluor Dyes (Invitrogen) for 1 h, coverslips were mounted onto glass slides using Slowfade Gold antifade mountant with DAPI (Invitrogen) to counterstain the DNA. Images were observed under fluorescence microscopy.

### Western blot

The distal colon tissues were lysed and the protein extract was quantified by BCA protein assay kit (Thermo Scientific). Equal whole tissue protein (50 μg) was run on SDS-PAGE gels (12.5%), followed by transfer to polyvinylidene fluoride membrane (Millipore, MA, USA). After blocked with 5% nonfat milk, the membrane was incubated with primary antibodies against β-actin (sc-8432, Santa Cruz Biotechnology) and CitH3 (ab5103, Abcam) overnight at 4°C. Finally, the membrane was incubated with HRP-conjugated secondary antibodies and the bands were scanned by Amersham Imager 600 ECL system (GE Healthcare; Chicago, IL, USA).

### Statistical analysis

Data were expressed as mean ± SEM and analyzed with GraphPad Prism 7 (GraphPad Software; La Jolla, CA, USA). Statistical comparisons were performed with paired or unpaired 2-tailed Student *t* test or one-way analysis of variance (ANOVA). Statistical significance was defined as follows: *p < .05, **p < .01, ***p < .001 and ****p < .0001.

## Results

### Butyrate markedly inhibits production of proinflammatory cytokines in neutrophils from patients with IBD

**Figure 1.**
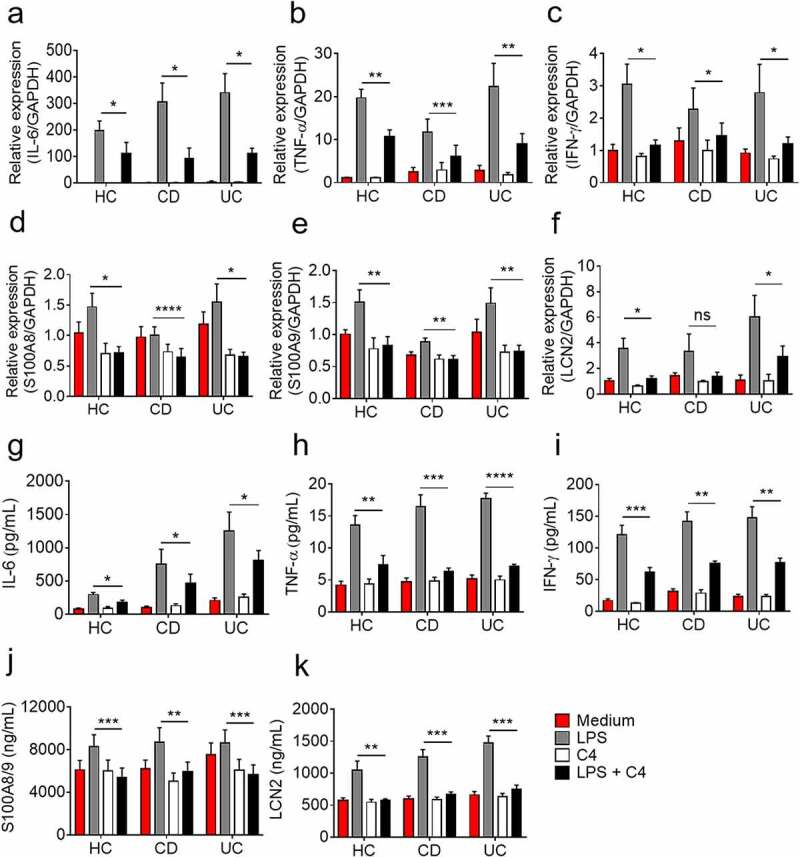
Butyrate inhibits neutrophils to produce proinflammatory cytokines, calprotectin and LCN2. Neutrophils were isolated from peripheral blood of healthy controls (HC, n = 8), patients with active CD (n = 10) and patients with active UC (n = 13) and were stimulated with LPS (300 ng/mL) in the presence or absence of C4 (0.5 mM) for 3 h. Cells were then collected and the levels of mRNA expression were analyzed for IL-6 (a), TNF-α (b), IFN-γ (c), S100A8 (d), S100A9 (e) and LCN2 (f), respectively, by qRT-PCR. Gene expression was normalized to GAPDH. Peripheral neutrophils (2 × 10^6^) were isolated from healthy controls (HC, n = 7), patients with active CD (n = 7) and patients with active UC (n = 7) and were stimulated with LPS (300 ng/mL) in the presence or absence of C4 (0.5 mM) for 24 h, and the culture supernatants were then collected for detection of IL-6 (d), TNF-α (e), IFN-γ (f), S100A8/9 (j) and LCN2 (k) by ELISA. *p < .05, **p < .01, ***p < .001 and ****p < .0001; ns, not significant. Data are representative of 3 independent experiments

Although neutrophils, as terminally differentiated myeloid-derived cells, have a weak de novo protein synthetic abilities,^19^ massive infiltration of neutrophils in inflamed intestine during colitis could produce and release a plethora of inflammatory mediators which contribute to the amplification of gut inflammation. We sought to investigate the impact of butyrate on the production of proinflammatory mediators from IBD patients. To this end, we isolated neutrophils from healthy controls and patients with active IBD and measured the production of inflammatory mediators. As shown in (Figure 1a-c), the mRNA levels of proinflammatory cytokines (e.g., IL-6, TNF-α, and IFN-γ) were markedly downregulated by butyrate treatment. The release of calprotectins (S100A8 and S100A9) and LCN2, defined as markers of relapse of IBD, was also remarkably suppressed by butyrate (Figure 1d-f). Consistently, the protein levels of IL-6, TNF-α, IFN-γ, S100A8/9, and LCN2 in the culture supernatants were also dramatically decreased (Figure 1g-k). Furthermore, neutrophil production of IL-17A and IL-22 was also observed to be decreased by butyrate treatment (Supplementary Figure S4a-b). Taken together, these results indicate that the short chain fatty acid butyrate markedly inhibits proinflammatory cytokine production from neutrophils of IBD patients.

### Butyrate suppresses production of neutrophil-derived chemokines and myeloperoxidase

**Figure 2.**
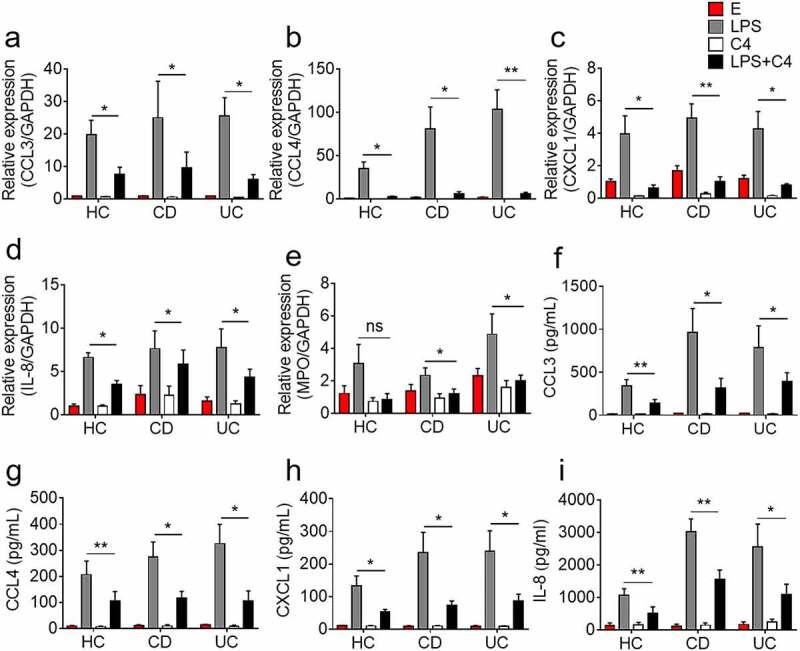
Butyrate inhibits production of chemokines and MPO by neutrophils. Neutrophils were isolated from peripheral blood of healthy controls (HC, n = 8), patients with active Crohn’s disease (CD, n = 10) and patients with active ulcerative colitis (UC, n = 13) and stimulated *in vitro* with LPS (300 ng/mL) in the presence or absence of C4 (0.5 mM) for 3 h. Cells then were collected and the levels of mRNA expression were analyzed for CCL3 (a), CCL4 (b), CXCL1 (c), IL-8 (d) and MPO (e), respectively, by qRT-PCR. Gene expression was normalized to GAPDH. Peripheral neutrophils (2 × 10^6^) were isolated from healthy donors (HC, n = 7), patients with active CD (n = 7) and patients with active UC (n = 7) and stimulated *in vitro* with LPS (300 ng/mL) in the presence or absence of C4 (0.5 mM) for 24 h, and the culture supernatants were collected for detection of CCL3 (f), CCL4 (g), CXCL1 (h), IL-8 (i) by ELISA. *p < .05 and **p < .01; ns, not significant. Data are representative of 3 independent experiments

It has been shown that various chemokines released from activated neutrophils further activate and recruit monocytes, macrophages and other adaptive immune cells to perpetuate inflammation. We next examined whether butyrate has an effect on neutrophil production of chemokines. (Figure 2a-d) shows that upon butyrate treatment, the levels of neutrophil-derived chemokines including CCL3, CCL4, CXCL1, and IL-8 were significantly decreased. Moreover, expression of CCL19, CCL20, and CXCL9 was also observed to be decreased by butyrate (Supplementary Figure S4c-e). Since over-production and release of myeloperoxidase (MPO) and reactive oxygen species (ROS) are confirmed to have a direct effect on tissue damage, we then asked whether butyrate has an effect on MPO production. As expected, butyrate was demonstrated to powerfully inhibit neutrophil production of MPO (Figure 2e). Moreover, the culture supernatants were also harvested and the levels of CCL3, CCL4, CXCL1, and IL-8 were determined by ELISA. As expected, butyrate markedly restrained neutrophils to produce these chemokines (Figure 2f-i).

### Butyrate inhibits neutrophil production of proinflammatory mediators through HDAC inhibitory function

**Figure 3.**
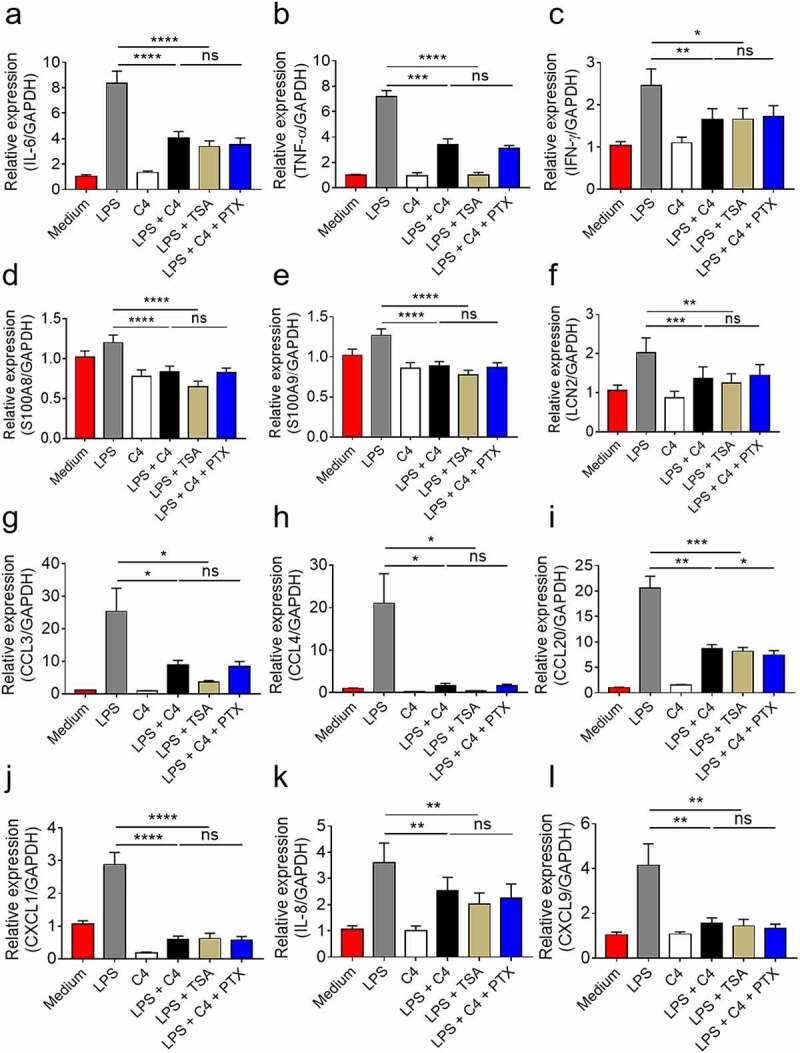
Butyrate inhibits neutrophil production of proinflammatory mediators in a HDACi-dependent manner. Neutrophils were isolated from peripheral blood of healthy donors (HC, n = 8) and stimulated *in vitro* with LPS (300 ng/mL) in the presence or absence of C4 (0.5 mM), TSA (10 μM) and PTX (100 ng/mL), respectively, for 3 h. The mRNA levels of proinflammatory mediators including IL-6 (a), TNF-α (b), IFN-γ (c), S100A8 (d), S100A9 (e), LCN2 (f), CCL3 (g), CCL4 (h), CCL20 (i), CXCL1 (j), IL-8 (k), and CXCL9 (l) were detected by qRT-PCR. Gene expression was normalized to GAPDH. *p < .05, **p < .01, ***p < .001 and ****p < .0001; ns, not significant. Data are representative of 3 independent experiments

Given that SCFAs function through two major pathways (i.e., inhibition of HDAC and signaling via GPCRs),^20^ we sought to investigate whereby butyrate exerts its effects. We then used two antagonists, a pan HDAC inhibitor (i.e., TSA) and GPCR inhibitor (i.e., PTX) to explore the intrinsic mechanisms of butyrate. Intriguingly, we found that TSA recapitulated the role of butyrate, whereas an addition of PTX in the presence of butyrate did not alter the inhibitory effects of butyrate, suggesting the effect of butyrate on production of proinflammatory mediators is mediated in a HDACi-dependent manner but not through GPCR signaling (Figure 3a-i).

### Butyrate suppresses neutrophil migration in vitro and weakens the formation of NETs

**Figure 4.**
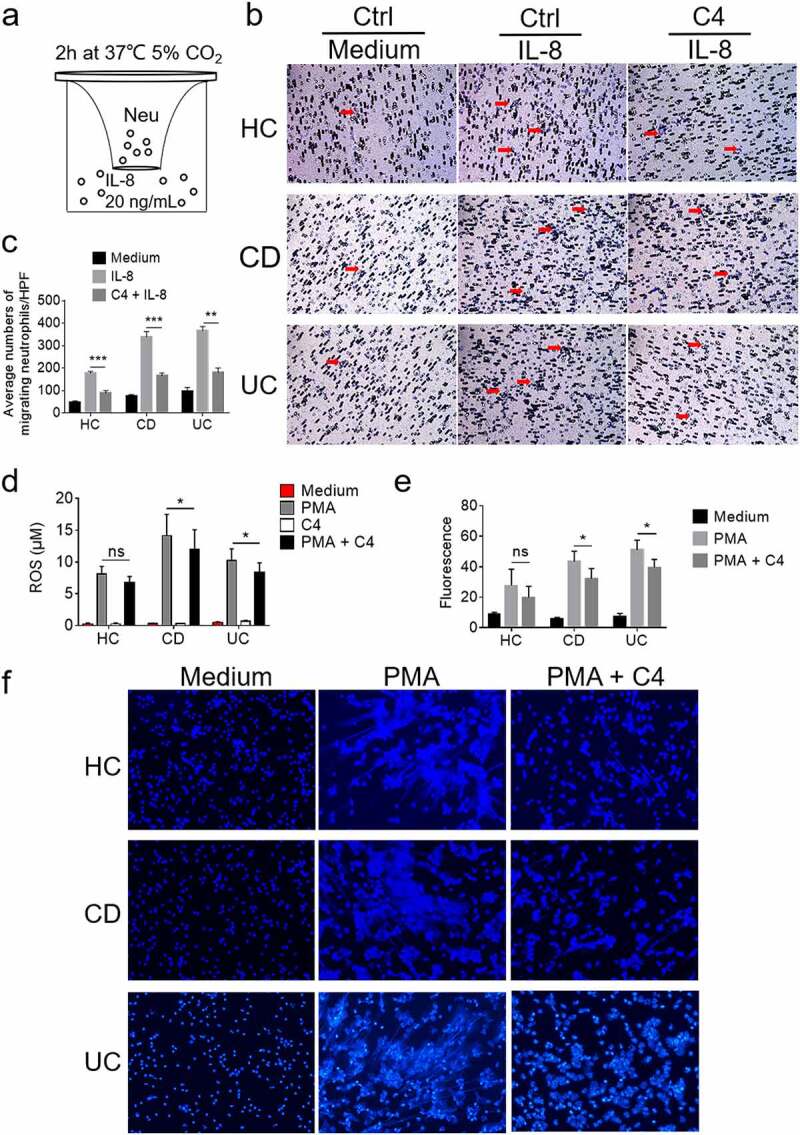
Butyrate suppresses migration and weakens the abilities of neutrophils to release ROS and NETs. Peripheral neutrophils (5 × 10^5^) were isolated from peripheral blood of healthy donors (HC, n = 6), patients with active Crohn’s disease (CD, n = 10) and patients with active ulcerative colitis (UC, n = 10) and seeded on the upper chamber of a Transwell insert. IL-8 (20 ng/mL) was loaded into the lower chamber to attract neutrophils. (a) Schematic representation of neutrophil migration *in vitro* for 2 h at 37°C. (b) Representative images of migrated neutrophils on the lower membrane stained with 0.1% crystal violet and observed under optical microscopy. Original magnification: ×200. (c) The histogram represents the number of migrating neutrophils per high-power field (HPF). (d) Peripheral neutrophils (1.5 × 10^4^) were isolated from peripheral blood of healthy donors (HC, n = 6), patients with active Crohn’s disease (CD, n = 10) and patients with active ulcerative colitis (UC, n = 10) and preincubated with or without C4 (0.5 mM) for 2 h, and then stimulated with PMA (100 ng/mL) to detect the production of ROS. The levels of ROS were measured by using Amplex Red Hydrogen Peroxide Assay Kit according to manufacturer’s instructions. (e, f) Peripheral neutrophils (5 × 10^5^) were isolated from peripheral blood of healthy donors (HC, n = 6), patients with active Crohn’s disease (CD, n = 8) and patients with active ulcerative colitis (UC, n = 8) and seeded on coverslips which were coated with poly-L-lysine. Adherent neutrophils were stimulated with PMA (100 ng/mL) in the presence or absence of C4 (10 mM) for 3 h at 37°C. Histograms of fluorescence intensity of NETs detected by fluorometric plate reader (e). Representative images of NETs were obtained from a HC (upper panels), a patient with A-CD (middle panels) and a patient with A-UC (lower panels) (f). Original magnification: ×200. *p < .05, **p < .01 and ***p < .001; ns, not significant. Data are representative of 3 independent experiments

Rapidly trafficking to the sites of inflammation is the first step and prerequisite of neutrophils to utilize their antimicrobial arsenal of effector mechanisms and then eradicate invasive pathogens. However, excessive recruitment and accumulation of neutrophils in inflamed mucosa are associated with increased intestinal permeability and mucosal injury. Therefore, we sought to figure out the effects of butyrate on the transmigration of neutrophils. To this end, neutrophils were isolated from patients with IBD and healthy donors and preincubated with or without butyrate for 30 minutes. Neutrophils were then seeded in the upper chamber for 2 h at 37°C with 5% CO_2_ to detect neutrophil migration toward lower chamber (Figure 4a). Neutrophils displayed robust migration to the bottom chamber in response to IL-8, whereas this effect was suppressed by coincubation with butyrate (Figure 4b,c).

Following the onset of inflammation, transmigrated neutrophils firstly mount on an antimicrobial attack via the phagocytosis and degranulation. In addition, the release of NETs, which are characterized by decondensed chromatin filaments coated with granule proteins, represents another pivotal antimicrobial strategy of neutrophils.^21^ In spite of their crucial role in host defense, a mountain of evidence shows that aberrant and prolonged release of NETs contributes to a variety of inflammatory or autoimmune disorders including systemic lupus erythematosus (SLE),^22^ vasculitis,^23^ and IBD.^24^ Previous study has demonstrated NET formation is dependent on ROS generated by NADPH oxidase.^25^ We firstly determined the role of butyrate in regulating PMA-induced ROS production. As expected, butyrate inhibited ROS production in neutrophils from IBD patients (Figure 4d). We then investigated whether butyrate has an inhibitory effect on the formation of NETs. In the absence of any stimuli, butyrate did not have any ability to induce the formation of NETs (data not shown). On the contrary, PMA-induced formation of NETs was indeed weakened by butyrate (Figure 4e,f), consistent with previous study showing that SCFAs attenuate the generation of NETs.^26^

### Butyrate suppresses the expression of genes related to inflammatory responses and leukocyte migration

**Figure 5.**
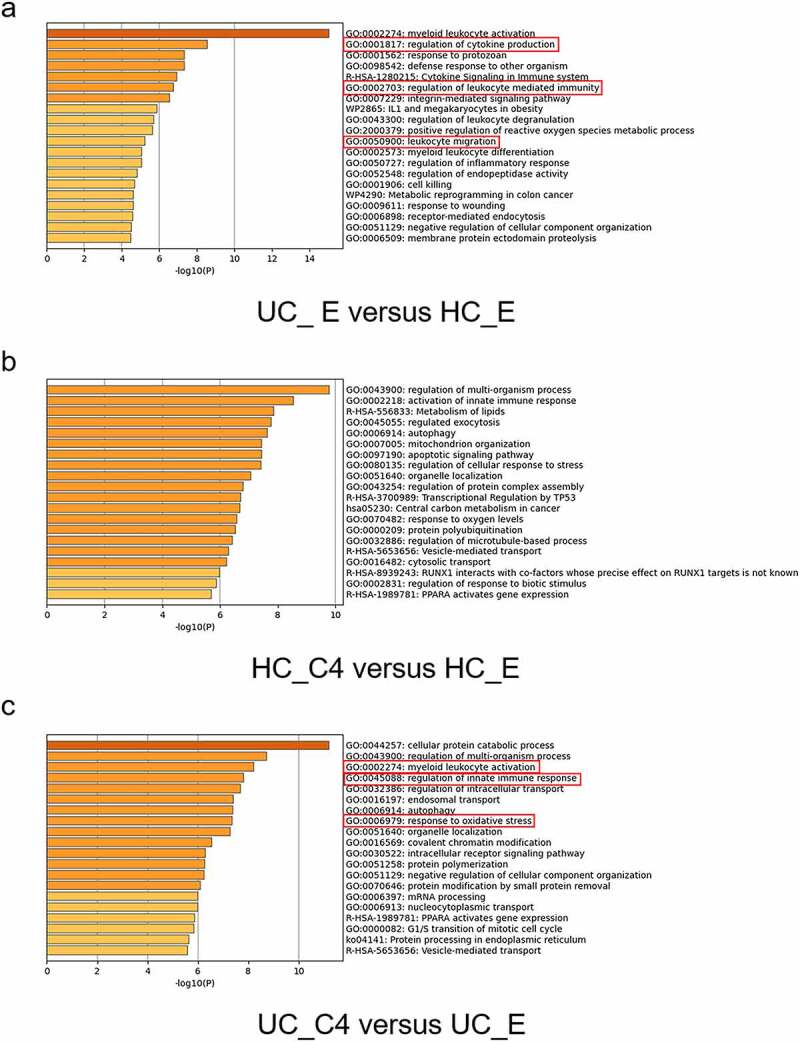
RNA sequencing analysis elicits the immunomodulatory effects of butyrate on IBD neutrophils. Neutrophils were isolated from healthy donors and patients with UC and treated *in vitro* with or without C4 (0.5 mM) for 3 h. The total RNA from four groups was extracted and performed with RNA sequencing to detect transcriptome differences. Differentially expressed genes (fold change > 2.0 or < 0.5 and *p* < .01) between two groups were enriched for GO functional analysis by Metascape. Top 10 pathways from the indicated comparisons are shown. (a-c) n = 3 biologically independent samples per group. HC_E, HC neutrophils treated without C4; HC_C4, HC neutrophils treated with C4; UC_E, UC neutrophils treated without C4; and UC_C4, UC neutrophils treated with C4

Based on the aforementioned results, we identified that butyrate has an inhibitory effect on neutrophil functions from both healthy donors and IBD patients. We then determined the dose-responses of butyrate on regulating neutrophils. As shown in (Supplementary Figure S5), the inhibitory effects of butyrate on neutrophil expression of proinflammatory mediators, ROS, and NETs appeared in a dose-dependent manner. RNA sequencing was then conducted and determined the differentially expressed genes under stimulation with butyrate. As shown in (Figure 5a), freshly isolated neutrophils from UC patients showed an increase of ability in immune response, cytokine production, and leukocyte migration compared to those from healthy controls. Butyrate-treated neutrophils from both groups exhibited significantly differentially expressed genes compared to untreated cells (Supplementary Figures S6,7). The pathway enrichment analysis further implicated that the functional differences were mainly reflected in leukocyte activation, regulation of innate immune response, and response to oxidative stress (Figure 5b,c). Twenty genes related to innate immune response were identified to be regulated by butyrate and heatmap was depicted in (Supplementary Figure S8). Collectively, these data confirm the *in vitro* role of butyrate in regulating IBD neutrophils and further suggest an immune-modulatory potential of butyrate in the pathogenesis of IBD.

### Oral administration of butyrate ameliorates the severity of DSS-induced murine colitis via inhibition of NET formation

**Figure 6.**
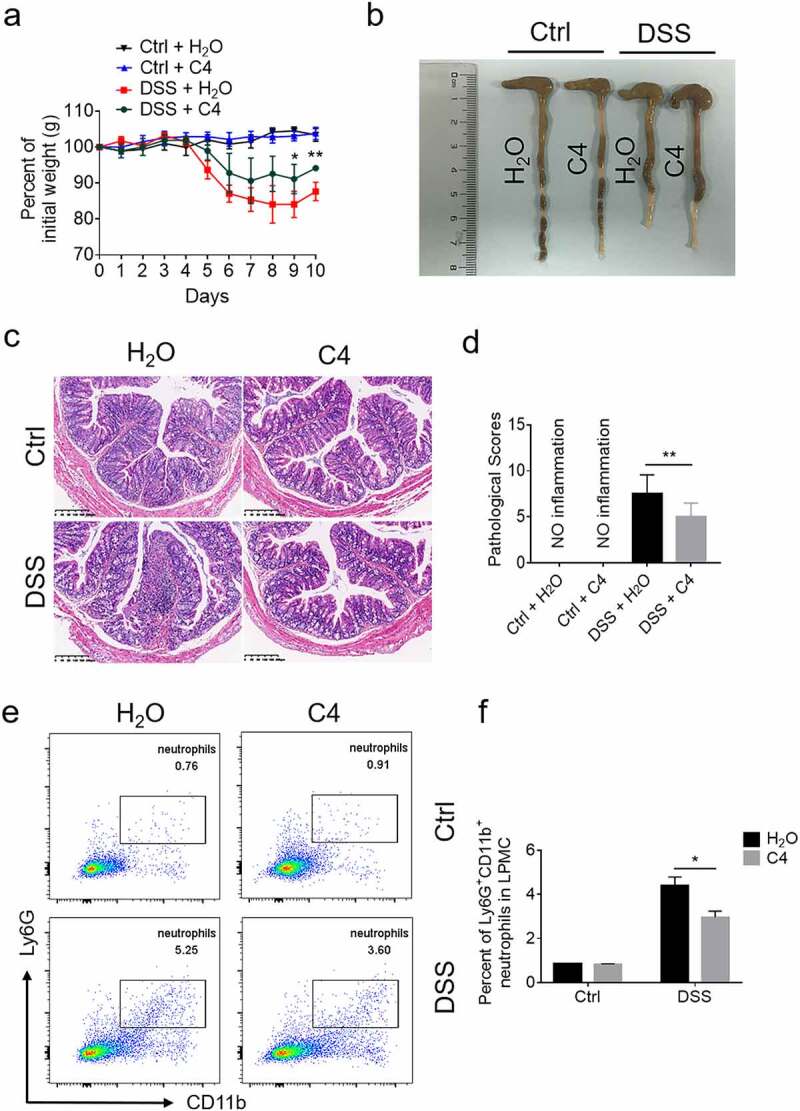
Oral administration of butyrate reduces neutrophil infiltration and protects from colitis induced by DSS. WT mice (n = 12 in each group) were administered 2% DSS in drinking water for 7 days followed by DSS free water for another 3 days. Mice from one of DSS-treated group were fed with C4 (200 mM) in drinking water from day 0 when DSS was given. (a) Changes of body weight during a period of 10-day observation. (b) Gross morphology of the colons on day 10 when mice were sacrificed. (c) Representative images of distal colonic sections after hematoxylin and eosin (H&E) staining. Scale bars: 200 μm. (d) Pathological scores of colon sections were calculated as indicated. (e) Lamina propria mononuclear cells (LPMC) were collected from colons of mice and flow cytometric analysis was performed to examine the percentages of CD11b^+^Ly6G^+^ neutrophils infiltrated in whole colon from each group. (f) Percentages of Ly6G^+^CD11b^+^ neutrophils in LPMC were shown in the bar chart. *p < .05 and **p < .01. Data are representative of 3 independent experiments

**Figure 7.**
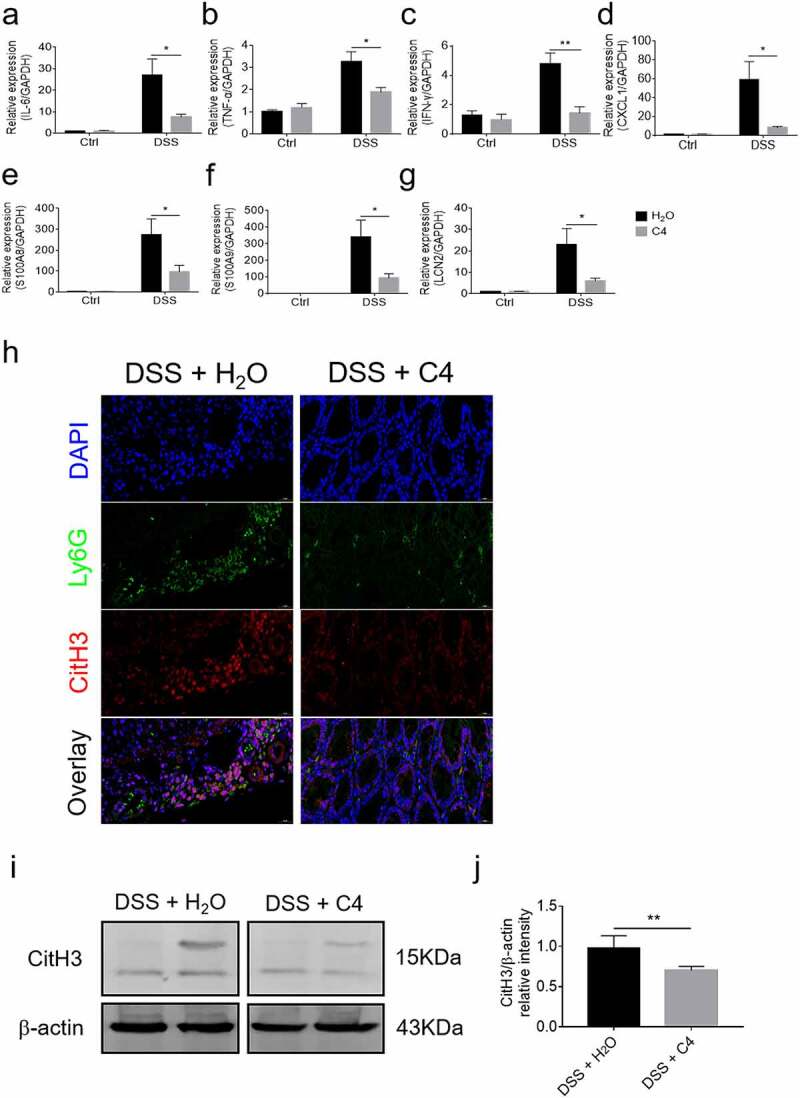
Butyrate inhibits NET formation and neutrophil-associated inflammatory mediators in DSS-induced murine colitis. Colon tissues were obtained from mice as described in Figure 6 on day 10 and total RNA was extracted to examine the mRNA levels of IL-6 (a), TNF-α (b), IFN-γ (c), CXCL1 (d), S100A8 (e), S100A9 (f) and LCN2 (g), respectively, by qRT-PCR. Gene expression was normalized to GAPDH. (h) Representative immunofluorescent images for detection of Citrullinated H3 (CitH3). Scale bars: 20 μm. (i, j) Lysates of the colon tissues were prepared and the protein levels of CitH3 were determined by Western blot with β-actin as a reference. *p < .05 and **p < .01. Data are representative of 3 independent experiments

In order to determine whether butyrate could prevent colitis development via regulating functions of neutrophils, colitis model was induced by administration of DSS as described above. Mice were treated with DSS in drinking water for 7 days followed with DSS-free water for additional 3 days. Colon tissues were collected for further analysis on sacrifice day. As shown in (Figure 6a-d), mice fed with butyrate displayed less severe disease, as exemplified by less weight change, longer colon length and lower levels of histological scores after DSS exposure. Besides, flow cytometric analysis revealed that the number of neutrophils infiltrated in the colons was markedly decreased from butyrate-treated mice compared to vehicle control (Figure 6e,f). Infiltration of CD4^+^ T cells and F4/80^+^ macrophages was decreased in intestinal mucosa of butyrate-treated colitic mice. In addition, the unfixed fresh cryosections of the distal colon tissues were used to detect the expression of ROS by dihydroethidium (DHE) staining. We found that the levels of ROS were decreased in butyrate-treated group, which were supposed to be ascribed to a reduced infiltration of inflammatory cells (Supplementary Figure S9a-b). As expected, the mRNA levels of IL-6, TNF-α, IFN-γ, CXCL1, S100A8, S100A9, and LCN2 were also markedly decreased in the colons of butyrate-treated mice compared with controls (Figure 7a-g). Moreover, we verified a marked decrease of CitH3, indicative of the formation of NETs in colon tissue sections from butyrate-treated mice in comparison to controls (Figure 7h-j). Collectively, these data suggest that butyrate ameliorates DSS-induced colitis at least partially through reduction of neutrophil-associated inflammatory mediators and inhibition of NET formation.

## Discussion

Host-microbial cross-talk plays an essential role in the maintenance of intestinal homeostasis. The intestinal tract acts as a reservoir for a vast diversity of microorganisms, and the ability to discriminate commensal, opportunistic and pathogenic microbes, and optimize an appropriate host response is essential for keeping a delicate and exquisite equilibrium in intestinal-microbial ecosystem. The normal gut commensal microbiota functions in different ways to confer beneficial functions, such as to help in nutrient metabolism, provide energy to the host, inhibit colonization of pathogens and maintain epithelial integrity and immunomodulation. Gut dysbiosis or an altered gut microbiota communities followed with destruction of host-microbial mutualism has been recognized as the defining event in the development of IBD.^27,28^ Epidemiological studies have shown loss of butyrate-producing bacterial species such as *Faecalibacterium prausnitzii*^29^ and *Roseburia hominis*,^30^ together with decreased levels of SCFAs^31^ in the feces from individuals with IBD compared to healthy donors. Immune-modulatory effect of butyrate on colitis has been reported elsewhere,^32^ whereas the exact roles in different immune cells such as neutrophils are not completely understood. In the current study, we demonstrated the critical role of butyrate in regulating functions of neutrophils from IBD patients and confirmed its *in vivo* role in preventing the development of DSS-induced colitis in mice.

Neutrophils are evolutionarily derived from the bone marrow as immediately responsive immune cells, rapidly migrate to the sites of intestinal inflammation and eradicate pathogens through a series of mechanisms including the phagocytosis, degranulation and release of extracellular traps. The exact role of neutrophils during intestinal inflammation is still elusive although both beneficial and detrimental functions have been reported.^18^ For example, depletion of neutrophils significantly exacerbates TNBS- or CD4^+^CD45RB^hi^ T cell transfer-induced colitis in mice.^16,33^ Furthermore, individuals with dysfunction of neutrophils display IBD-like colitis.^34^ Our previous study has depicted a subset of neutrophils (i.e., CD177^+^ neutrophils) to have a protective role in the maintenance of mucosal integrity.^15^ Moreover, our recent work also demonstrated a novel effect of cyclosporine A on neutrophils, showing that cyclosporine A functions as fueling glycolysis and aerobic oxidation to restrain excessive activation, contributing to the induction of clinical remission in acute severe UC patients.^35^ In contrast, inflamed mucosa from patients with active IBD is infiltrated with large numbers of neutrophils, and the degree of neutrophil infiltration is thought to be instrumental for the assessment of disease activity.^17^

Neutrophil-derived cytokines and chemokines play an important role in the initiation and perpetuation of inflammation.^19^ These inflammatory mediators mediate intercellular cross-talk to boost local immune responses in gut mucosa. In our current study, we found that neutrophil-derived proinflammatory cytokines and chemokines were significantly inhibited by butyrate. Calprotectin, heterodimer of S100A8 and S100A9, constitutes approximately 40% of the cytosolic proteins of neutrophils,^36^ which is reported to contribute to the pathogenesis of IBD. Elevated concentrations of calprotectin in stool and plasma are detected in patients with active IBD and correlate well with disease activity.^37^ In addition, abnormally high levels of ROS in inflamed mucosa and concomitant imbalance between oxidants and antioxidants cause oxidative DNA damage to intestinal epithelial cells in IBD.^38^ Interestingly, we found that butyrate-treated neutrophils produced low levels of S100A8, S100A9, ROS, and MPO. Further analysis by using TSA and PTX revealed that butyrate inhibited neutrophil production of proinflammatory mediators in a HDACi-dependent manner.

Transmigrated neutrophils are considered a hallmark of inflammatory conditions and mediate an impairment of epithelial barrier function and tissue damage through the release of inflammatory mediators and oxidative substances. Reappearance of neutrophils in intestinal mucosa from IBD patients with clinical remission has also been considered as a hallmark of disease relapse, and the decrease and absence of mucosal neutrophils is a goal of histological healing.^39^ To illustrate the influence of butyrate on the migration of neutrophils, we utilized a Transwell model and identified that butyrate could inhibit IL-8-induced neutrophil transmigration.

NETs are web-like decondensed DNA network decorated with different cytosol and granule proteins, which are released by highly activated neutrophils to entrap microbes^40^ and prevent dissemination.^41^ In spite of immune-protective roles of NETs during infectious status, accumulating lines of evidence have suggested that dysregulated NETs cause immunopathology in different inflammatory diseases like SLE, psoriasis, and cystic fibrosis.^42,43^ NETs are found to accumulate in inflamed intestinal mucosa from patients with active UC and sustain inflammatory signals.^44^ Recent studies also demonstrated the pathological role of NETs in inflamed mucosa, such as impairing intestinal barrier function,^45^ and inducing intestinal damage and thrombotic tendency.^24^ PMA is an artificial stimuli to profoundly induce neutrophil production of extracellular traps. Previous studies have demonstrated that both autophagy and superoxide generation are essential for the formation of NETs.^46^ As mentioned above, butyrate could inhibit neutrophil generation of ROS, and significantly suppressed the formation of NETs. RNA sequencing analysis further revealed the differentially expressed gene profiles of butyrate-treated neutrophils and confirmed the immune-modulatory effects on IBD neutrophils. We thus conducted DSS-induced murine colitis to ascertain the *in vivo* role of butyrate in modulating the functions of neutrophils. In line with *in vitro* studies, butyrate-treated mice exhibited less severe disease characterized by decreased infiltration of neutrophils and associated inflammatory mediators. Moreover, NETs in inflamed colon were also decreased by butyrate treatment. Given the decrease of NETs in inflamed intestinal mucosa,^47^ it is supposed to be attributable to the effects of butyrate on neutrophils.

To date, the exact role of neutrophils in the induction of intestinal inflammation is still controversial, being both beneficial and detrimental as reported in previous studies. Mice depleted with neutrophils suffer severe disease in DSS-induced colitis,^48,49^ and lysophosphatidylserine (LysoPS) released by apoptotic neutrophils promotes ILC3 production of IL-22, leading to promoting tissue repair in DSS-induced colitis.^48^ These data suggest that neutrophils participate in tissue repair and the maintenance of mucosal homeostasis. However, excessive accumulation of neutrophils and release of proinflammatory mediators indeed exacerbate colitis.^18^ The key points of resolution of intestinal inflammation may lie on limitation of proinflammatory responses in acute phase and promotion elimination of neutrophils through apoptosis or efferocytosis in chronic phase. In addition to the beneficial effects of butyrate on limitation of proinflammatory potential of neutrophils, amelioration of colitis by butyrate may also be associated with promoting CD4^+^ T cell production of IL-10 and IL-22,^8,10^ boosting macrophage antimicrobial program^9^ and strengthening epithelial barrier integrity.^4^

Collectively, our study ascertains that butyrate inhibits proinflammatory potential of neutrophils, thus ameliorating mucosal inflammation in IBD. However, recent studies have observed the detrimental role of butyrate in inflammation-induced loss of epithelial barrier function,^50^ consistent with the finding showing that butyrate mediates inhibition of colonic growth via HDACi.^51^ This could be one of the reasons in that butyrate is not widely used yet and still stuck in clinical trial stage in medication of IBD. Acute recruitment of neutrophils into inflamed mucosa helps to resolve inflammation whereas chronic infiltration exacerbates and perpetuates mucosal inflammation in the gut. Therefore, our data suggest that butyrate may be used clinically as auxiliary medication in acute phase of intestinal inflammation to limit further recruitment and activation of immune cells rather in chronic phase when epithelial proliferation and repair may be impeded.

In summary, the present study shows that butyrate has a direct immune-modulatory effect on neutrophils from patients with IBD and plays a protective role in DSS-induced colitis. Our study identifies a critical role of butyrate in modulating the functions of neutrophils through a reduced production of proinflammatory mediators, inhibition of migration and formation of NETs, thus contributing to restraining intestinal inflammation (Supplementary Figure S10). Therefore, these results provide a novel therapeutic avenue via modulating neutrophil function by butyrate in the maintenance of intestinal homeostasis and control of gut inflammation.

## Supplementary Material

Supplemental Material

## Figures and Tables

**Table:** 

Butyrate	C4
CCL	C-C motif chemokine ligand
CD	Crohn’s disease
CXCL	C-X-C motif chemokine ligand
GPCR	G protein-coupled receptor
HDAC	histone deacetylase
HC	healthy controls
IBD	inflammatory bowel disease
IFN-γ	interferon gamma
IL	interleukin
ILC	innate lymphoid cells
LCN2	lipocalin 2
LPS	lipopolysaccharide
MPO	myeloperoxidase
NETs	neutrophil extracellular traps
PMA	phorbol 12-myristate 13-acetate
PTX	pertussis toxin
ROS	reactive oxygen species
S100A8	S100 calcium-binding protein A8
S100A9	S100 calcium-binding protein A9
SCFAs	short chain fatty acids
SLE	systemic lupus erythematosus
TNF-α	tumor necrosis factor alpha
TSA	trichostatin A
UC	ulcerative colitis
