# Supplementary material for: Microbiota metabolite butyrate constrains neutrophil functions and ameliorates mucosal inflammation in inflammatory bowel disease
Source: Gut Microbes. 2021 Sep 8;13(1):1968257. doi: 10.1080/19490976.2021.1968257 (PMC8437544; doi:10.1080/19490976.2021.1968257)
Supplement: Supplemental Material [file KGMI_A_1968257_SM1878.zip › Supplementary information/Supplementary figure legends.docx]

**Supplementary Figure Legends**

**Supplementary Figure 1.** The purity of isolated peripheral neutrophils. Peripheral neutrophils were isolated from anticoagulated whole blood with Ficoll-Paque gradient centrifugation. After hypotonic lysis of red blood cells, total cells were used for analysis of the purity of neutrophils by flow cytometry (≥ 95%).

**Supplementary Figure 2.** Different concentrations of butyrate do not affect the survival of neutrophils. Neutrophils were isolated from anticoagulated whole blood of healthy donor (HC, n=3). After *in vitro* stimulation with different concentrations of butyrate for periods as indicated, neutrophils were collected and stained with live/dead dye and fluorochrome-conjugated anti-human antibodies against CD66b, and the survival of neutrophils was evaluated by means of flow cytometry. The number in each panel indicates the percentage of live neutrophils. Data are representative of 3 independent experiments.

**Supplementary Figure 3.** Different concentrations of butyrate used in this study do not affect apoptosis of neutrophils. Neutrophils were isolated from anticoagulated whole blood of healthy donors (HC, n=3). After stimulation with indicated concentrations of butyrate for 3 h, neutrophils were collected and stained with Annexin V-FITC and PI, and apoptosis was evaluated by means of flow cytometry. TNF-α (20 ng/mL) was used to induce neutrophil apoptosis as a positive control. The Annexin V-FITC^+^ cells in each panel indicates the apoptotic neutrophils. Data are representative of 3 independent experiments.

**Supplementary Figure 4.** Butyrate inhibits production of neutrophil-derived cytokines and chemokines. Neutrophils were isolated from peripheral blood of healthy donors (HC, n=8), patients with active CD (n=10) and patients with active UC (n=13) and stimulated *in vitro* with LPS (300 ng/mL) in the presence or absence of butyrate (C4) (0.5 mM) for 3 h. Cells were then collected and the levels of mRNA expression were analysed for IL-17A (a), IL-22 (b), CCL19 (c), CCL20 (d), CXCL1(e), respectively, by qRT-PCR. Gene expression was normalized to GAPDH. **p* < .05, ***p* < .01 and ****p* < .001; ns, not significant.

**Supplementary Figure 5.** Dose-responses of butyrate on neutrophil production of proinflammatory mediators, ROS and NETs. Neutrophils were isolated from anticoagulated whole blood of HC (n=7) and stimulated *in vitro* with indicated concentrations of butyrate ranging from 0.5 mM to 10 mM for 3 h in the presence or absence of LPS (300 ng/mL) or PMA (100 ng/mL). The production of proinflammatory mediators including IL-6 (a), TNF-α (b), IFN-γ (c), IL-22 (d), LCN2 (e), CCL3 (f), CCL4 (g), CCL20 (h), CXCL1 (i), IL-8 (j), ROS (k), and NETs (l) was determined as mentioned in **Materials and Methods**. **p* < .05, ***p* < .01 and ****p* < .001 versus neutrophils treated with LPS.

**Supplementary Figure 6.** Volcano plots of all genes in the indicated comparisons are shown with differentially expressed genes. Neutrophils isolated from HC (n=3) and UC patients (n=3) were stimulated *in vitro* in the presence or absence of C4 (0.5 mM) for 3 h. RNA sequencing was performed and the number of differentially expressed genes was shown in volcano plot.

**Supplementary Figure 7.** Heatmap shows row-scaled expression of differential gene expression. Neutrophils isolated from HC (n=3) and UC patients (n=3) were stimulated *in vitro* in the presence or absence of C4 (0.5 mM) for 3 h. RNA sequencing was performed and differentially expressed genes among four groups were shown in heatmap.

**Supplementary Figure 8.** Heatmap shows row-scaled expression of 20 innate immune response-associated genes regulated by butyrate in both HC and UC neutrophils. Neutrophils isolated from HC (n=3) and UC patients (n=3) were stimulated *in vitro* in the presence or absence of C4 (0.5 mM) for 3 h, and RNA sequencing was performed. After treatment with C4, differentially expressed genes related to the GO pathway regulation of innate immune response were depicted as heatmap.

**Supplementary Figure 9.** Numbers of CD4^+^ T cells and F4/80^+^ macrophages and ROS levels in colon tissues are decreased after butyrate treatment. (a) Colon tissues from DSS-induced colitic mice treated with or without butyrate as shown in Figure 6 were collected for immunohistochemical staining for CD4 and F4/80. Original magnification: ×200. (b) In addition, unfixed cryosections of distal colon tissues were incubated with fluorescein ROS indicators (Dihydroethidium, DHE) to detect ROS. The images from DSS-induced colitis mice treated with or without butyrate were observed by using fluorescence microscopy under the same exposure conditions. Original magnification: ×200. Data are representative of 3 independent experiments.

**Supplementary Figure 10.** Role of butyrate in regulating functions of neutrophils in IBD. Mucosal inflammation in IBD is characterized by disruption of epithelial barrier, large infiltration of different immune cells and proinflammatory mediators. Butyrate inhibits IBD neutrophils release of proinflammatory mediators such as IL-6, TNF-α, calprotectins and LCN2, which is mediated by a HDAC inhibition. Moreover, butyrate also suppresses neutrophil migration and release of ROS and NETs which further contribute to amplification of mucosal inflammation. Hence, butyrate regulation of neutrophil function may serve as a negative regulator in constraining mucosal inflammation.
