# Supplementary material for: Microbiota metabolite butyrate constrains neutrophil functions and ameliorates mucosal inflammation in inflammatory bowel disease
Source: Gut Microbes. 2021 Sep 8;13(1):1968257. doi: 10.1080/19490976.2021.1968257 (PMC8437544; doi:10.1080/19490976.2021.1968257)
Supplement: Supplemental Material [file KGMI_A_1968257_SM1878.zip › Supplementary information/Supplementary methods.docx]

**Supplementary Methods**

**Immunohistochemistry**

Samples of colon tissues from DSS-treated mice were collected, fixed with 4% paraformaldehyde and embedded in a paraffin block followed by cutting into 5 μm slices. These sections were then transferred onto glass slides for further use. After deparaffinization, rehydration and antigen retrieval, these slides were treated with 0.3% Triton X-100 for 10 min at room temperature and blocked with 10% normal donkey serum for 1 h. Subsequently, immunostaining was performed with rabbit anti-mouse CD4 (ab183685, Abcam) or rat anti-mouse F4/80 (ab100790, Abcam) overnight at 4℃. The slips were then treated with 3% H_2_O_2_ for elimination of endogenous peroxidase. After 3 washes with PBS, the slides were incubated with horseradish peroxidase-conjugated goat anti-rabbit secondary antibodies for 30 min. The color reaction was developed with 3,3’-diaminobenzidine, and the slides were counterstained with hematoxylin. Images were observed under optical microscopy.

**RNA-sequencing library preparation and transcriptome sequencing**

Neutrophils were isolated from healthy donors (n=3) and patients with UC (n=3), and treated with or without C4 (0.5 mM) for 3 h. The total RNA from four groups were extracted and performed with RNA-sequencing to detect transcriptome differences. RNA degradation and contamination were monitored on 1% agarose gels. RNA purity was checked using the NanoPhotometer spectrophotometer (IMPLEN, CA, USA) and integrity by using the RNA Nano 6000 Assay Kit of the Bioanalyzer 2100 system (Agilent Technologies, CA, USA). A total amount of 1 μg RNA per sample was used as input material for the RNA sample preparations. Sequencing libraries were generated using NEBNext® UltraTM RNA Library Prep Kit for Illumina® (NEB; Ipswich, MA, USA) following manufacturer’s recommendations and index codes were added to attribute sequences to each sample. The clustering of the index-coded samples was performed on a cBot Cluster Generation System using TruSeq PE Cluster Kit v3-cBot-HS (Illumina; San Diego, CA, USA) according to the manufacturer’s instructions. After cluster generation, the library preparations were sequenced on an Illumina Novaseq platform and 150 bp paired-end reads were generated.

**Transcriptomic data analysis**

Raw data (raw reads) of fastq format were firstly processed through in-house perl scripts. In this step, clean data (clean reads) were obtained by removing reads containing adapter, reads containing ploy-N and low-quality reads from raw data. At the same time, Q20, Q30 and GC content the clean data were calculated. All downstream analyses were based on the clean data with high quality. Reference genome and gene model annotation files were downloaded from genome website directly. Index of the reference genome was built using Hisat2 v2.0.5 and paired-end clean reads were aligned to the reference genome using Hisat2 v2.0.5. And the FPKM of each gene was then calculated based on the length of the gene and reads count mapped to this gene. Differential expression analysis was performed using the DESeq2 R package (1.16.1). Genes with an adjusted *P*-value <.05 found by DESeq2 were assigned as differentially expressed. For Gene Ontology (GO) and KEGG enrichment analysis, differentially expressed genes were implemented by the clusterProfiler (<https://bioconductor.org/packages/release/bioc/html/clusterProfiler.html>) and Metascape. Heat maps were generated using the pHeatmap R package (<https://CRAN.R-project.org/package=pheatmap>).
