# Supplementary material for: Microbiota metabolite butyrate constrains neutrophil functions and ameliorates mucosal inflammation in inflammatory bowel disease
Source: Gut Microbes. 2021 Sep 8;13(1):1968257. doi: 10.1080/19490976.2021.1968257 (PMC8437544; doi:10.1080/19490976.2021.1968257)
Supplement: Supplemental Material [file KGMI_A_1968257_SM1878.zip › Supplementary information/Supplementary tables.docx]

**Supplementary Tables**

**Supplementary Table 1.** Demographic and Clinical features of IBD patients

|  | Blood samples | | |
| --- | --- | --- | --- |
|  | HC | A-CD | A-UC |
| Number of patients | 32 | 37 | 43 |
| Age (years) | 39.7±14.4 | 33.2±12.5 | 41.0±15.4 |
| Gender (Female/Male) | 19/13 | 18/19 | 14/29 |
| Duration (months) | | 73.7±63.1 | 58.9±60.1 |
| Current therapy | | | |
| 5-ASA | | 20 | 31 |
| Glucocorticoids | | 5 | 11 |
| Immunosuppressants | | 7 | 1 |
| Biologics (infliximab) | | 3 | 4 |
| Disease extent^a^ | | | |
| E1  E2  E3 | | | 2 |
|  |  |  | 8 |
|  |  |  | 33 |
| Disease location^a^ | | | |
| L1 | | 10 |  |
| L2 | | 14 |  |
| L3 | | 13 |  |
| L4 | | 0 |  |
| Mayo score | |  | 8.0±2.4 |
| CDAI | | 221.1±60.1 |  |

^a^Montreal classification

**Supplementary Table 2.** The list of Primers used for qRT-PCR analyses

| Gene | Species | Forward Sequence (5’ to 3’) | Reverse Sequence (5’ to 3’) |
| --- | --- | --- | --- |
| GAPDH | Human | GGAGCGAGATCCCTCCAAAAT | GGCTGTTGTCATACTTCTCATGG |
| IL-6 | Human | ACTCACCTCTTCAGAACGAATTG | CCATCTTTGGAAGGTTCAGGTTG |
| IL-17A | Human | AGATTACTACAACCGATCCACCT | GGGGACAGAGTTCATGTGGTA |
| IL-22 | Human | ACAACACAGACGTTCGTCTCATTG | GAACAGCACTTCTTCAAGGGTGA |
| TNF-α | Human | GAGGCCAAGCCCTGGTATG | CGGGCCGATTGATCTCAGC |
| IFN-γ | Human | TCGGTAACTGACTTGAATGTCCA | TCGCTTCCCTGTTTTAGCTGC |
| S100A8 | Human | ATGCCGTCTACAGGGATGAC | ACTGAGGACACTCGGTCTCTA |
| S100A9 | Human | AAAAGGTCATAGAACACATCATGG | GAAGCTCAGCTGCTTGTCTG |
| MPO | Human | GAGCAGGACAAATACCGCACCA | AGAGAAGCCGTCCTCATACTCC |
| LCN2 | Human | GACAACCAATTCCAGGGGAAG | GCATACATCTTTTGCGGGTCT |
| CCL3 | Human | TCTGCATCACTTGCTGCTGACAC | CACTCAGCTCCAGGTCGCTGAC |
| CCL4 | Human | CTGTGCTGATCCCAGTGAATC | TCAGTTCAGTTCCAGGTCATACA |
| CCL19 | Human | CTGCTGGTTCTCTGGACTTCC | AGGGATGGGTTTCTGGGTCA |
| CCL20 | Human | TGCTGTACCAAGAGTTTGCTC | CGCACACAGACAACTTTTTCTTT |
| CXCL1 | Human | TTTTGAAATGTCAACCCCAAG | GATCTCATTGGCCATTTGCT |
| CXCL8 | Human | ACTGAGAGTGATTGAGAGTGGAC | AACCCTCTGCACCCAGTTTTC |
| CXCL9 | Human | CCAGTAGTGAGAAAGGGTCGC | AGGGCTTGGGGCAAATTGTT |
| GAPDH | Mouse | GGTTGTCTCCTGCGACTTCA | TGGTCCAGGGTTTCTTACTCC |
| TNF-α | Mouse | GGTGCCTATGTCTCAGCCTCTT | GCCATAGAACTGATGAGAGGGAG |
| IL-6 | Mouse | GATGGATGCTACCAAACTGGAT | CCAGGTAGCTATGGTACTCCAGA |
| IFN-γ | Mouse | ATGAACGCTACACACTGCATC | CCATCCTTTTGCCAGTTCCTC |
| CXCL1 | Mouse | CTGGGATTCACCTCAAGAACATC | CAGGGTCAAGGCAAGCCTC |
| S100A8 | Mouse | AAATCACCATGCCCTCTACAAG | AAATCACCATGCCCTCTACAAG |
| S100A9 | Mouse | ATACTCTAGGAAGGAAGGACACC | TCCATGATGTCATTTATGAGGGC |
| LCN2 | Mouse | TGGCCCTGAGTGTCATGTG | CTCTTGTAGCTCATAGATGGTGC |
