# Supplementary figures and images for: Microbiota metabolite butyrate constrains neutrophil functions and ameliorates mucosal inflammation in inflammatory bowel disease
Source: Gut Microbes. 2021 Sep 8;13(1):1968257. doi: 10.1080/19490976.2021.1968257 (PMC8437544; doi:10.1080/19490976.2021.1968257)

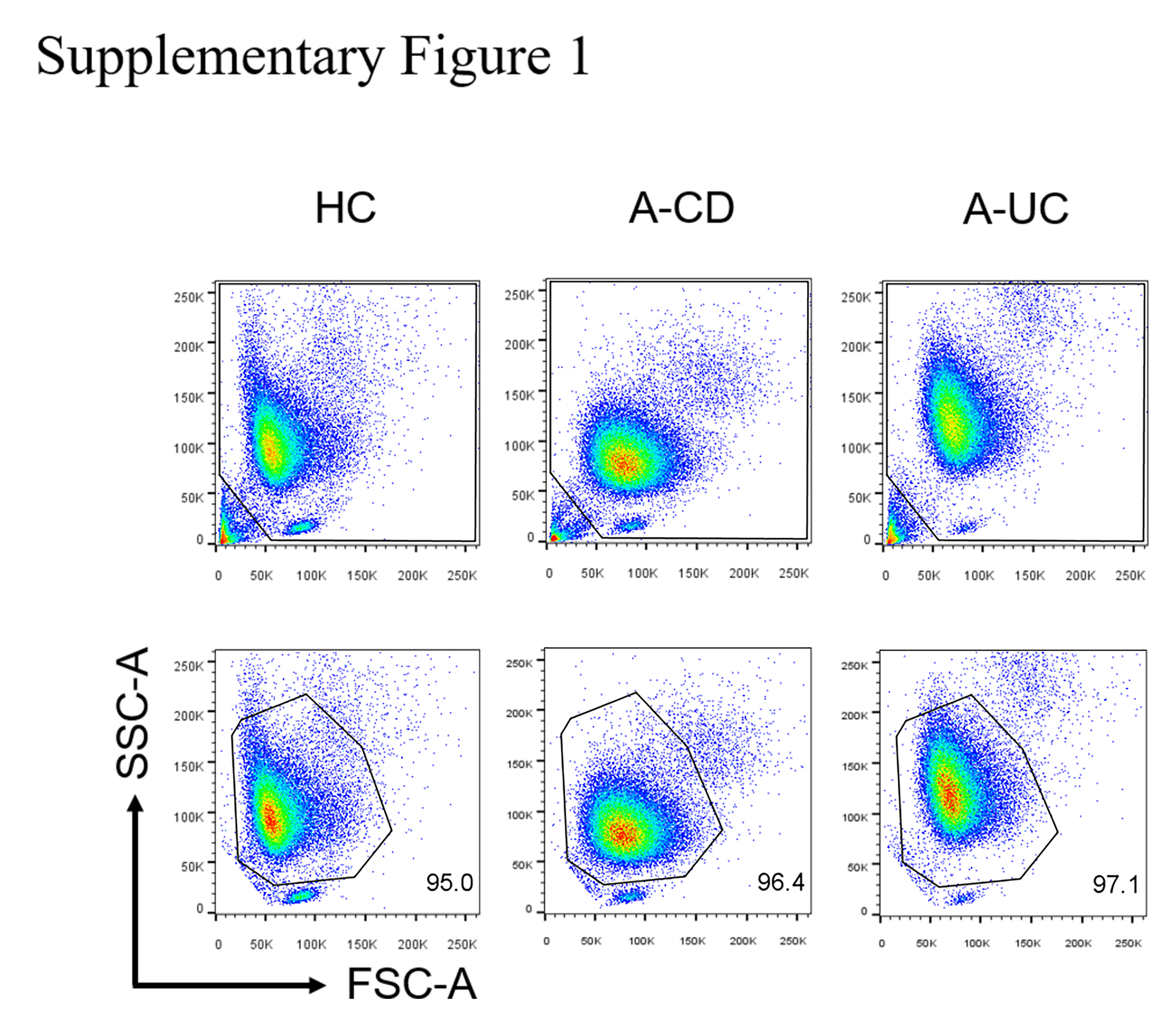

Supplement: Supplemental Material [file KGMI_A_1968257_SM1878.zip › Supplementary information/Supplementary Figure 1.tif]

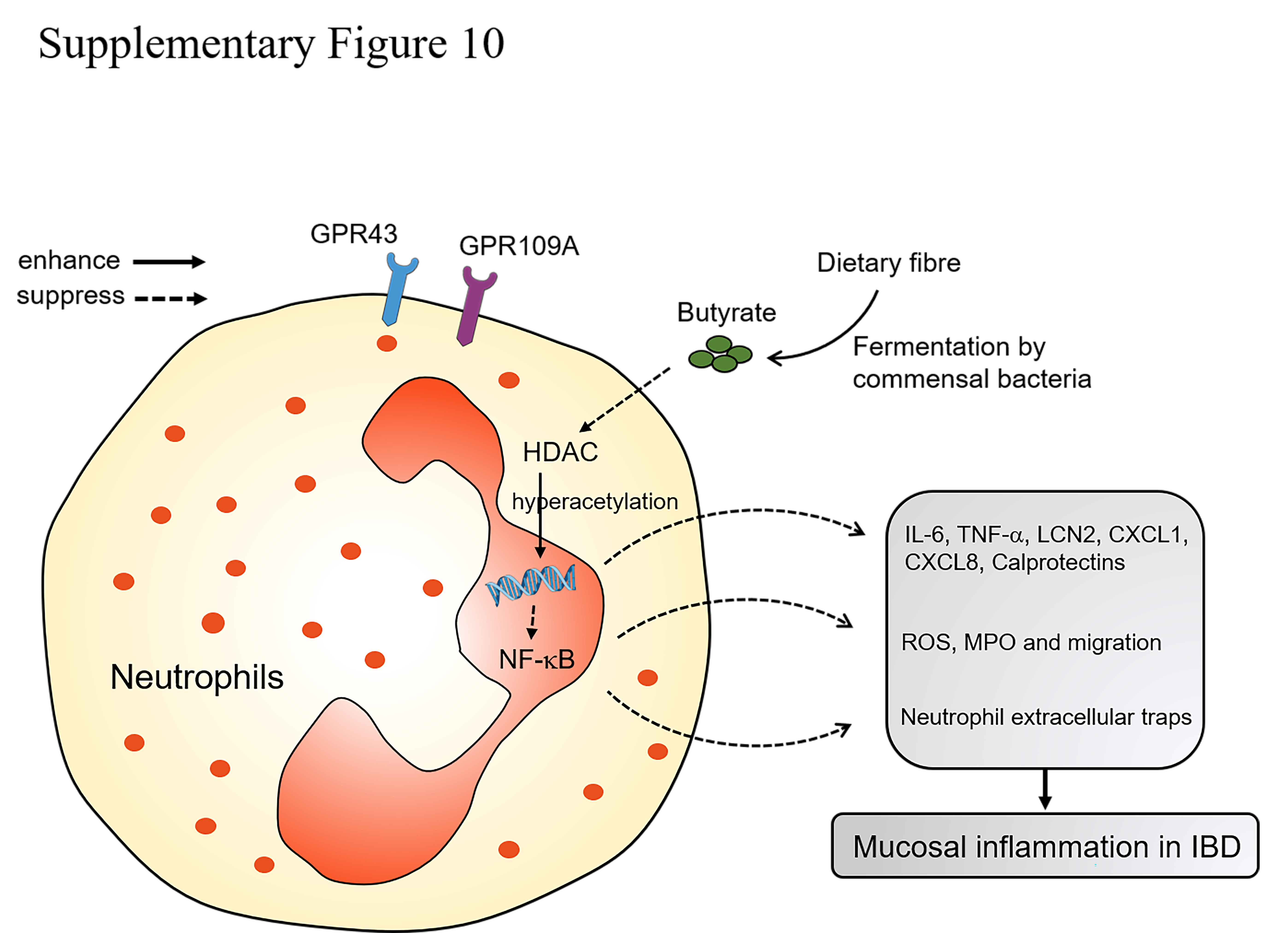

Supplement: Supplemental Material [file KGMI_A_1968257_SM1878.zip › Supplementary information/Supplementary Figure 10.tif]

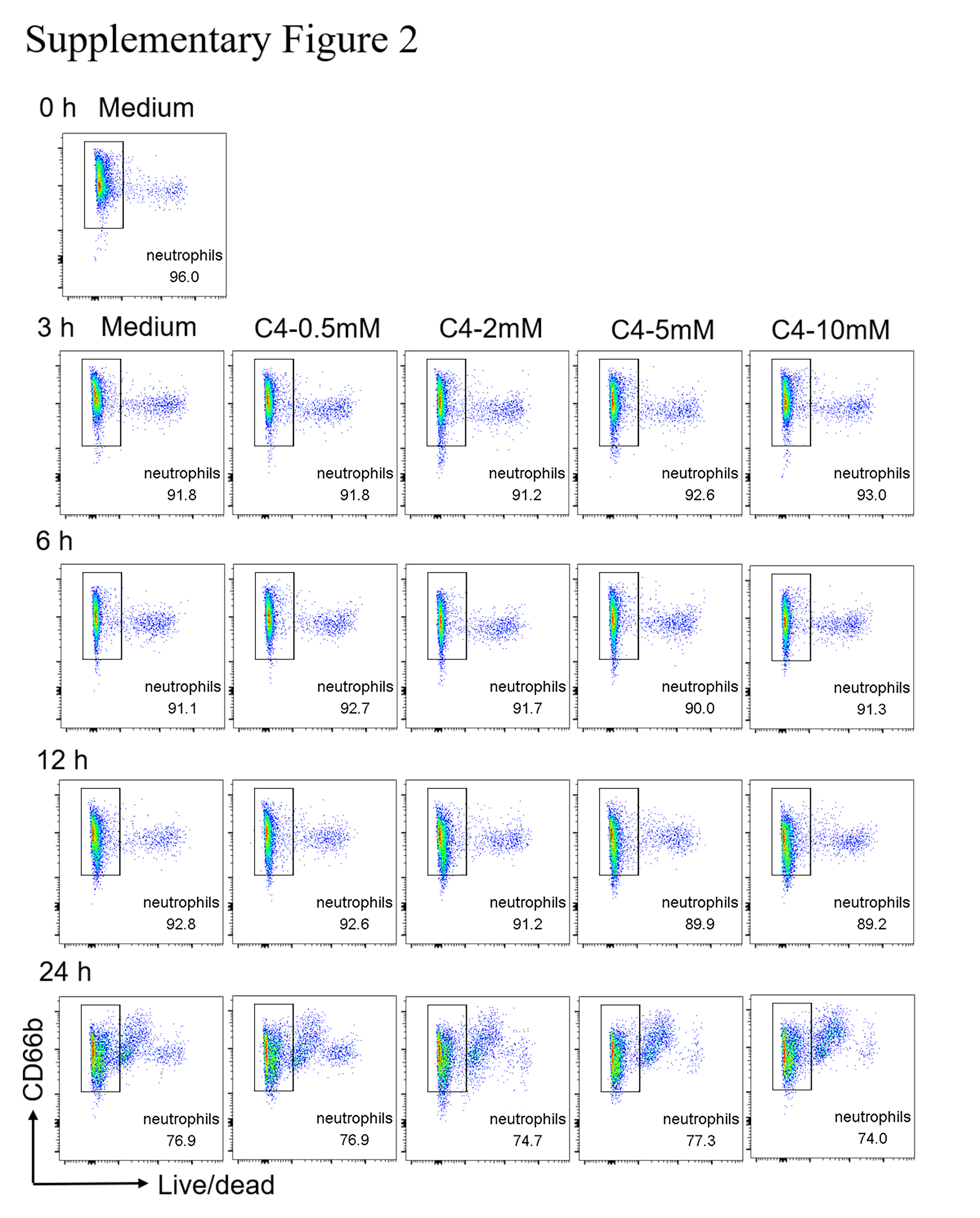

Supplement: Supplemental Material [file KGMI_A_1968257_SM1878.zip › Supplementary information/Supplementary Figure 2.tif]

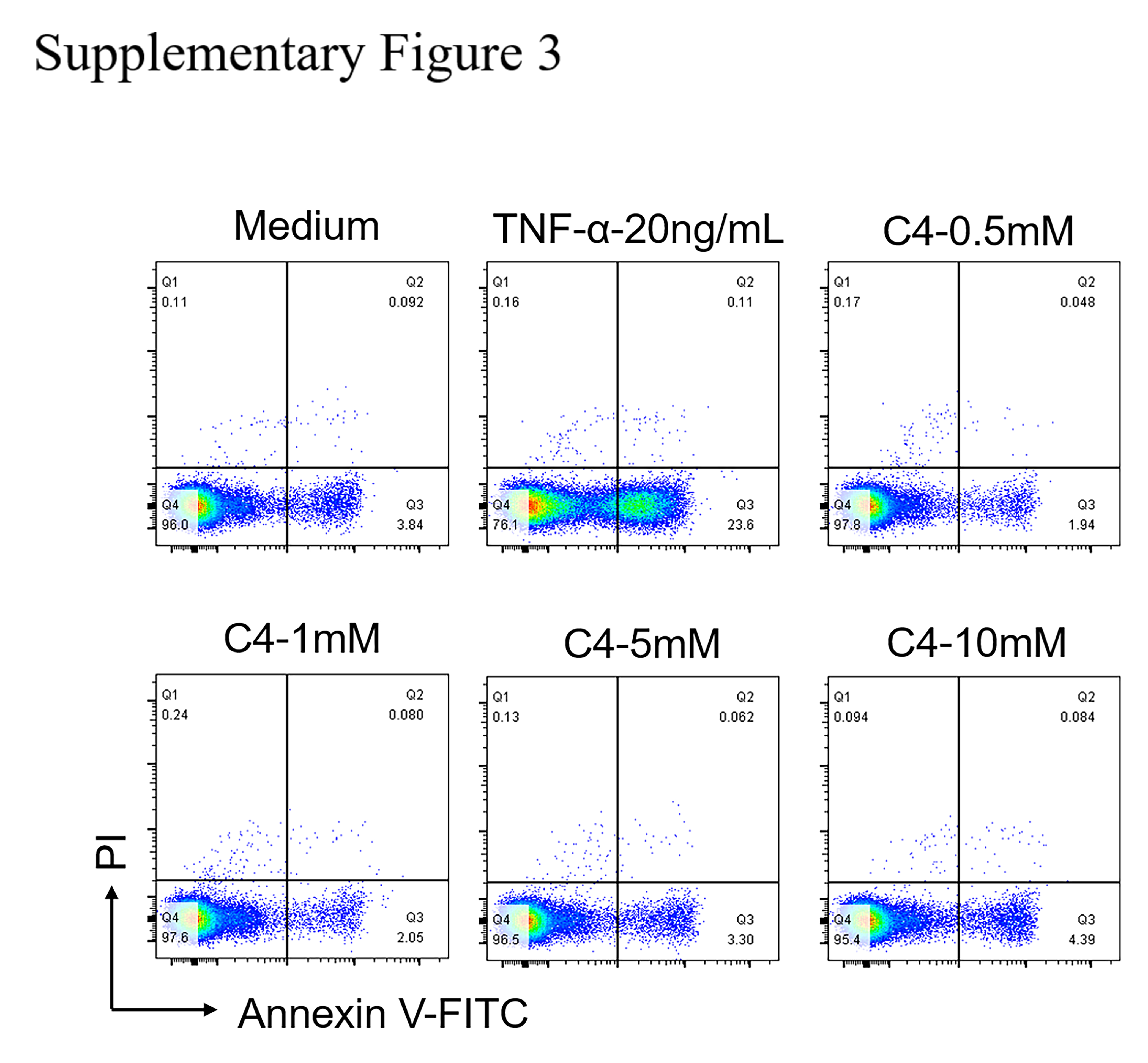

Supplement: Supplemental Material [file KGMI_A_1968257_SM1878.zip › Supplementary information/Supplementary Figure 3.tif]

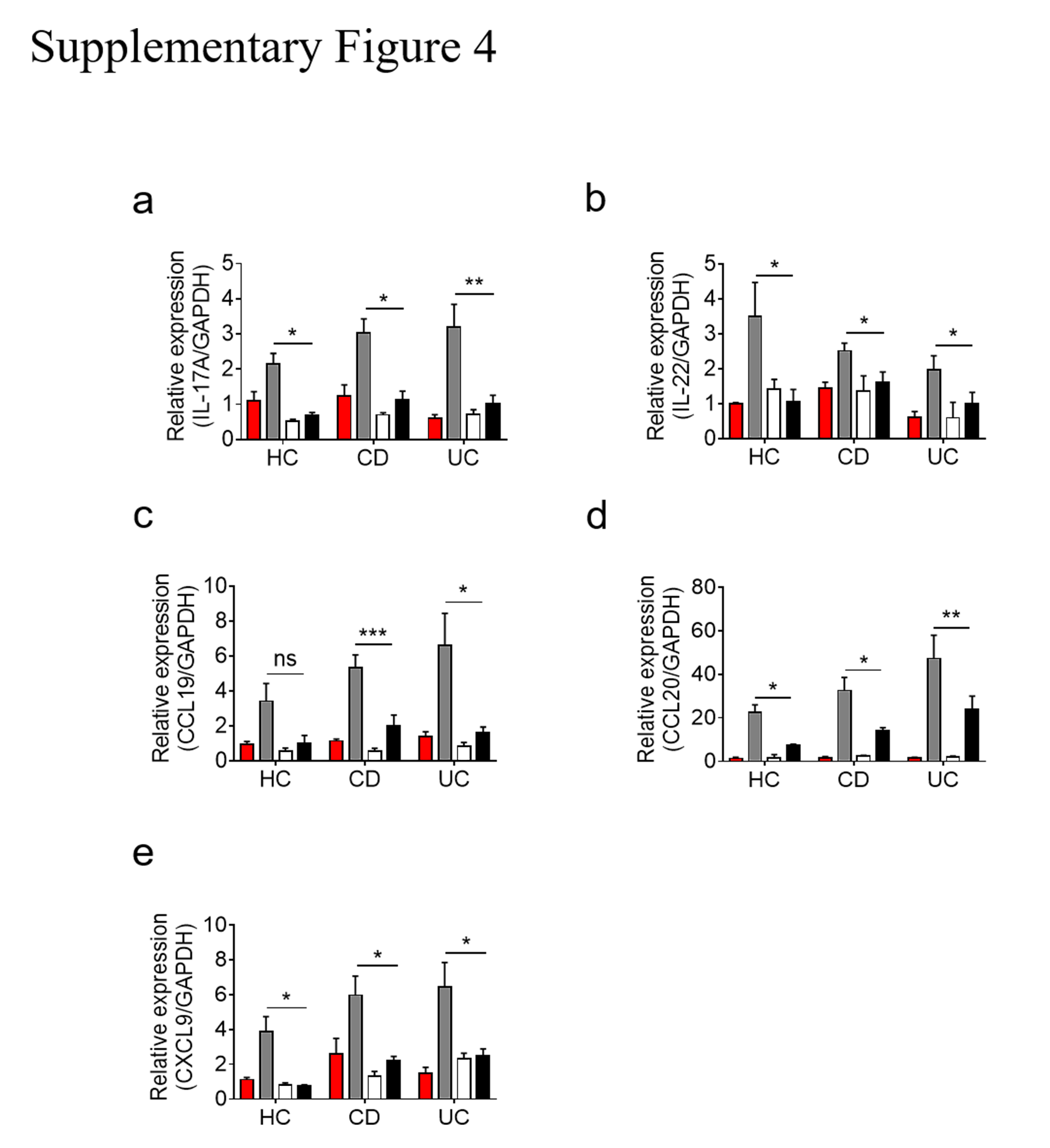

Supplement: Supplemental Material [file KGMI_A_1968257_SM1878.zip › Supplementary information/Supplementary Figure 4.tif]

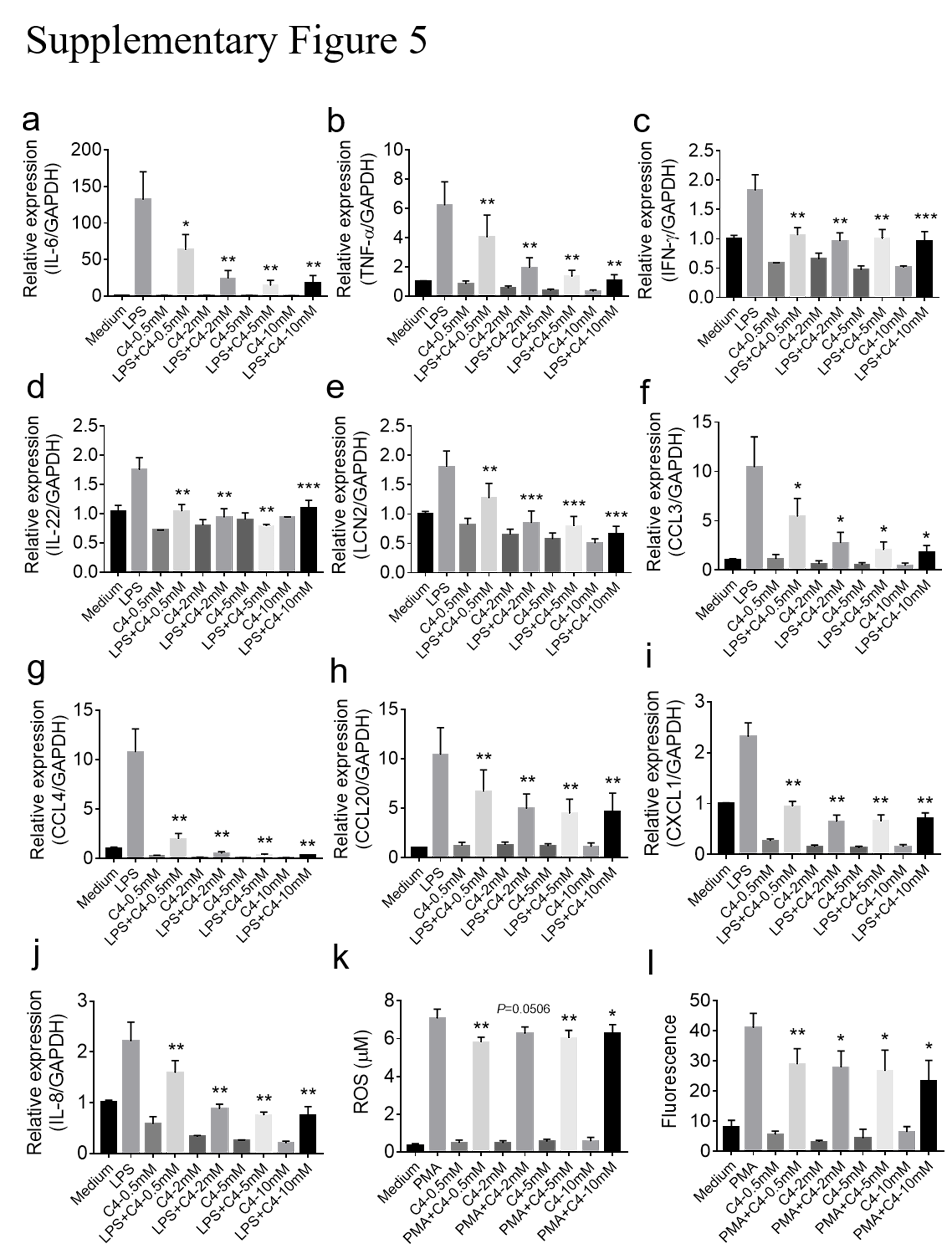

Supplement: Supplemental Material [file KGMI_A_1968257_SM1878.zip › Supplementary information/Supplementary Figure 5.tif]

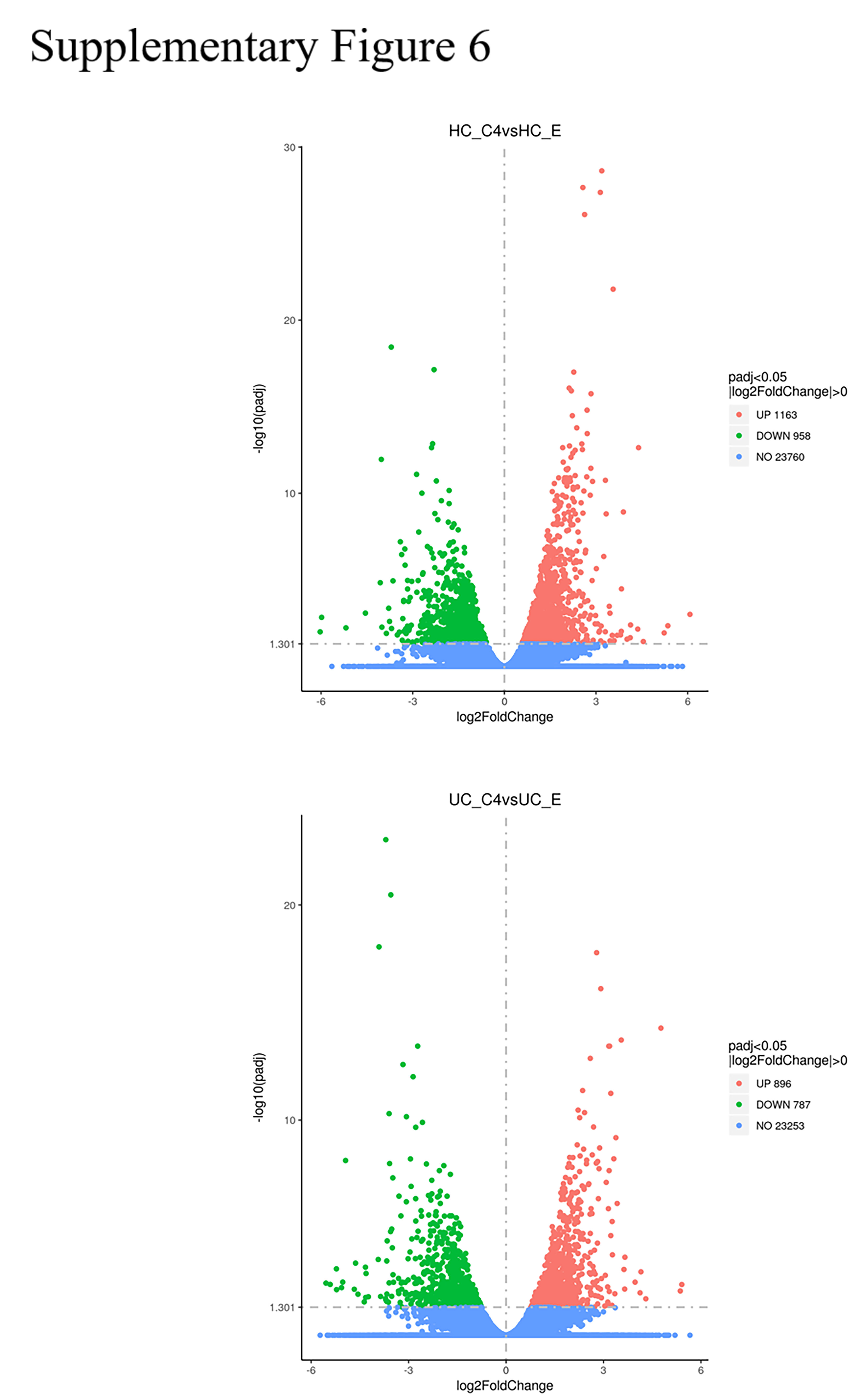

Supplement: Supplemental Material [file KGMI_A_1968257_SM1878.zip › Supplementary information/Supplementary Figure 6.tif]

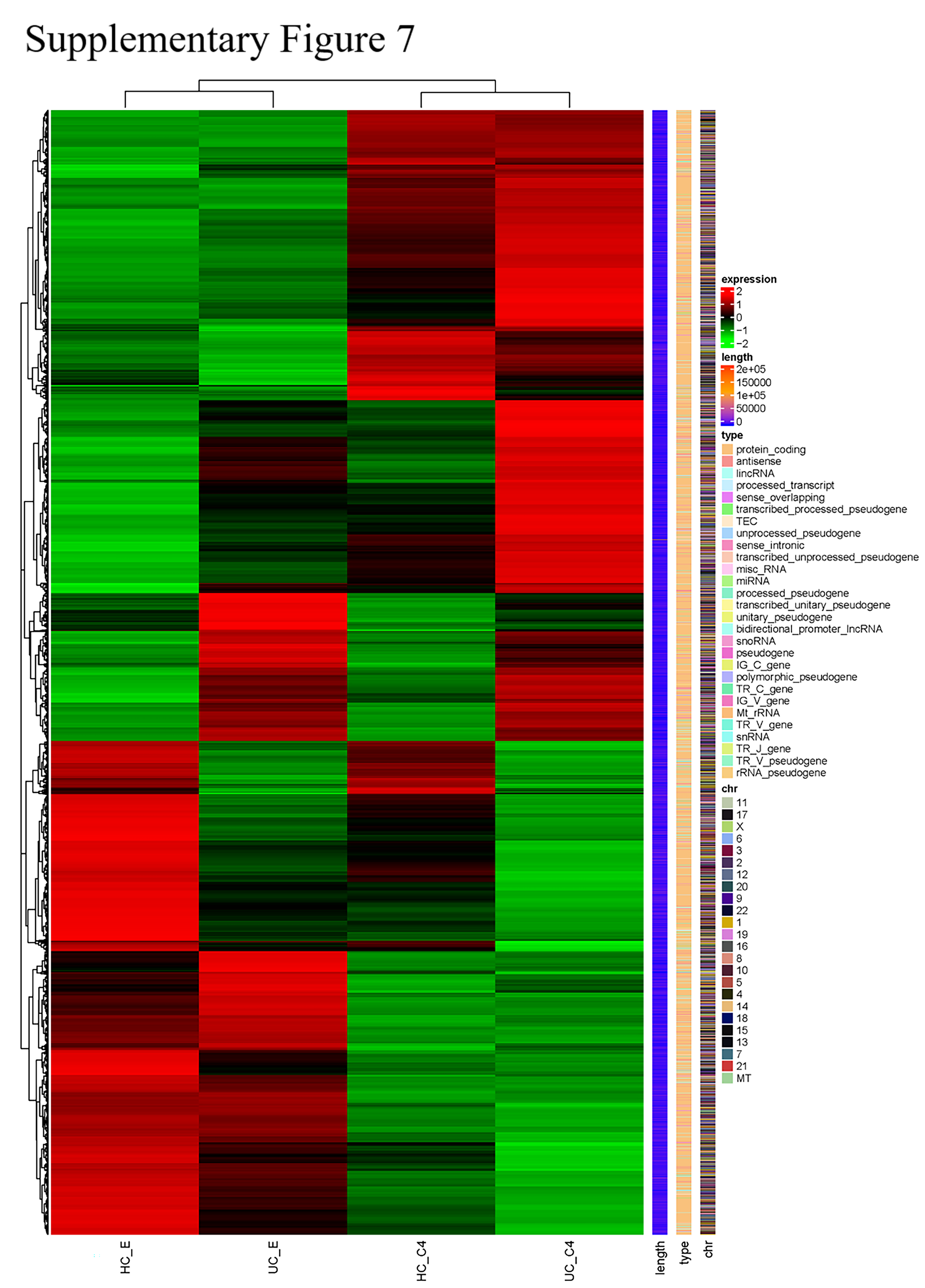

Supplement: Supplemental Material [file KGMI_A_1968257_SM1878.zip › Supplementary information/Supplementary Figure 7.tif]

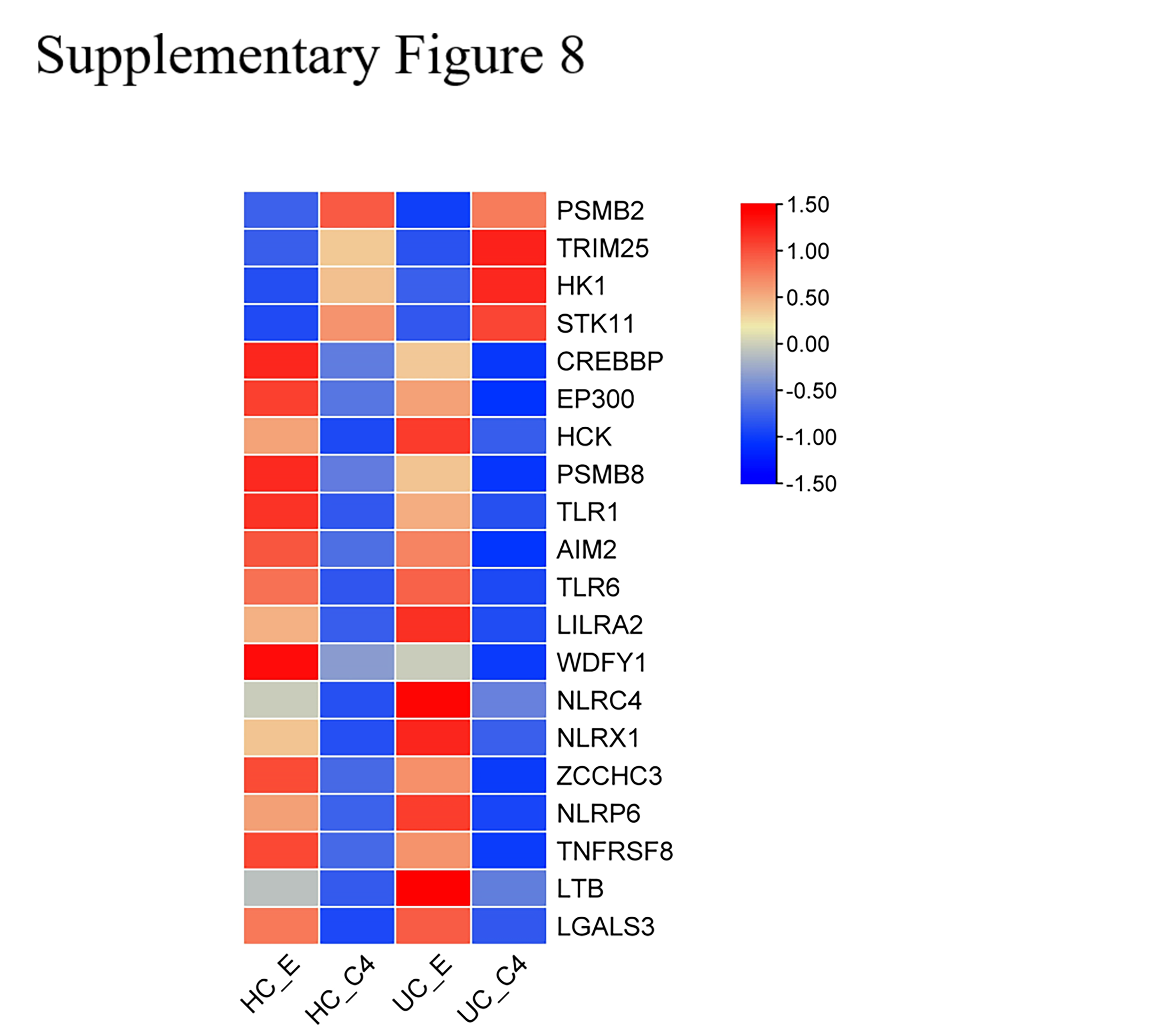

Supplement: Supplemental Material [file KGMI_A_1968257_SM1878.zip › Supplementary information/Supplementary Figure 8.tif]

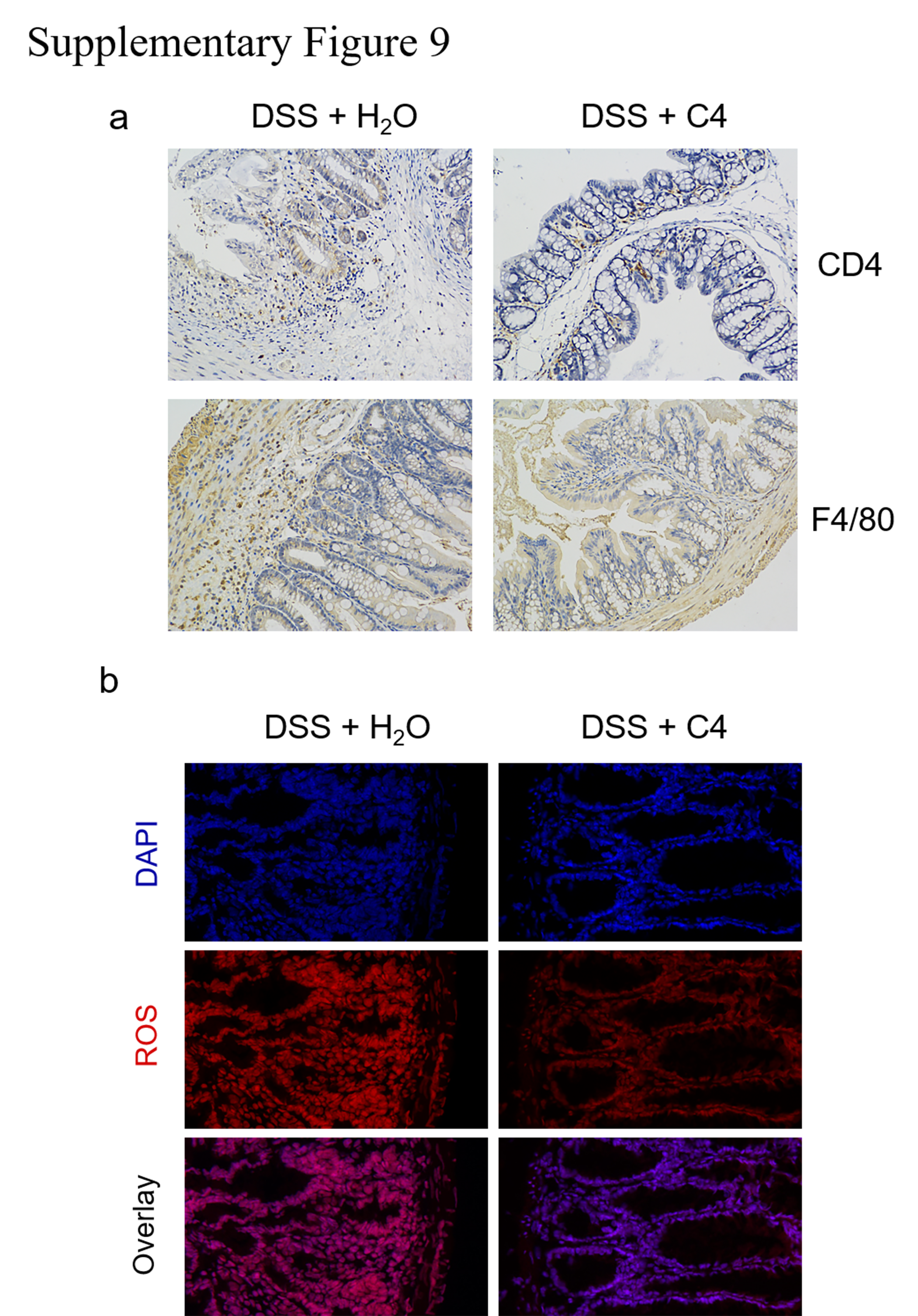

Supplement: Supplemental Material [file KGMI_A_1968257_SM1878.zip › Supplementary information/Supplementary Figure 9.tif]
